# Mycobactericidal Activity of Sutezolid (PNU-100480) in Sputum (EBA) and Blood (WBA) of Patients with Pulmonary Tuberculosis

**DOI:** 10.1371/journal.pone.0094462

**Published:** 2014-04-14

**Authors:** Robert S. Wallis, Rodney Dawson, Sven O. Friedrich, Amour Venter, Darcy Paige, Tong Zhu, Annette Silvia, Jason Gobey, Craig Ellery, Yao Zhang, Kathleen Eisenach, Paul Miller, Andreas H. Diacon

**Affiliations:** 1 Formerly Pfizer Inc, Groton, Connecticut, United States of America; 2 University of Cape Town, Cape Town, South Africa; 3 Division of Medical Physiology, Department of Biomedical Sciences, Faculty of Medicine and Health Sciences, Stellenbosch University, Cape Town, South Africa; 4 Medical Research Council Centre for Molecular and Cellular Biology, Department of Biomedical Sciences, Faculty of Medicine and Health Sciences, Stellenbosch University, Cape Town, South Africa; 5 Pfizer, Groton, Connecticut, United States of America; 6 University of Arkansas for Medical Sciences, Little Rock, Arkansas, United States of America; Faculty of Medicine, Australia

## Abstract

**Rationale:**

Sutezolid (PNU-100480) is a linezolid analog with superior bactericidal activity against *Mycobacterium tuberculosis* in the hollow fiber, whole blood and mouse models. Like linezolid, it is unaffected by mutations conferring resistance to standard TB drugs. This study of sutezolid is its first in tuberculosis patients.

**Methods:**

Sputum smear positive tuberculosis patients were randomly assigned to sutezolid 600 mg BID (N = 25) or 1200 mg QD (N = 25), or standard 4-drug therapy (N = 9) for the first 14 days of treatment. Effects on mycobacterial burden in sputum (early bactericidal activity or EBA) were monitored as colony counts on agar and time to positivity in automated liquid culture. Bactericidal activity was also measured in *ex vivo* whole blood cultures (whole blood bactericidal activity or WBA) inoculated with *M. tuberculosis* H37Rv.

**Results:**

All patients completed assigned treatments and began subsequent standard TB treatment according to protocol. The 90% confidence intervals (CI) for bactericidal activity in sputum over the 14 day interval excluded zero for all treatments and both monitoring methods, as did those for cumulative WBA. There were no treatment-related serious adverse events, premature discontinuations, or dose reductions due to laboratory abnormalities. There was no effect on the QT interval. Seven sutezolid-treated patients (14%) had transient, asymptomatic ALT elevations to 173±34 U/L on day 14 that subsequently normalized promptly; none met Hy's criteria for serious liver injury.

**Conclusions:**

The mycobactericidal activity of sutezolid 600 mg BID or 1200 mg QD was readily detected in sputum and blood. Both schedules were generally safe and well tolerated. Further studies of sutezolid in tuberculosis treatment are warranted.

**Trial Registration:**

ClinicalTrials.gov NCT01225640

## Introduction

*Mycobacterium tuberculosis* resistant to current first-line anti-tuberculosis agents is a serious and growing global health threat, causing at least 444,000 new tuberculosis (TB) cases and 150,000 deaths annually [1]. Treatments for drug-resistant (DR) TB are inferior to those for drug-sensitive disease, with lower cure rates, reduced safety and tolerability, and prolonged treatment requirements. Oxazolidinone antimicrobials are increasingly viewed as candidates for inclusion in new regimens for DR-TB, as they have a distinct mechanism of action (binding to the 23S ribosome, thereby blocking microbial protein synthesis) without cross-resistance to existing TB drugs. However, in the case of linezolid, the only currently licensed oxazolidinone, serious hematologic and neurologic toxicities arise during long term use due to inhibition of mitochondrial protein synthesis that often require dose reduction or discontinuation [2], [3]. Thus, for the promise of oxazolidinones for TB to be realized, new drugs in this class with superior efficacy and reduced toxicity must be developed.

Sutezolid (PNU-100480) is a thiomorpholinyl analog of linezolid with preliminary evidence for superior efficacy against *M. tuberculosis*. In the mouse model, sutezolid shortens standard treatment by 1 month, whereas linezolid does not [4]; in the whole blood culture model, the maximal bactericidal activity of sutezolid (−0.42 log/day) is more than twice that of linezolid (−0.16 log/day, P<0.001) [5]. Time-dependent killing has been reported in whole blood and hollow fibers [5], [6]. Bactericidal activity against intracellular mycobacteria is mainly due to the parent (PNU-100480), whereas a sulfoxide metabolite (PNU-101603) contributes significantly to activity against extracellular mycobacteria [6]. Phase 1 studies revealed no abnormal hematologic or biochemical findings, nor instances of peripheral or ophthalmic neuropathy, in healthy volunteers administered sutezolid 600 mg twice daily for 28 days [5], potentially indicating a safety profile superior to similarly dosed linezolid.

In the present study, sutezolid was administered at doses of 600 mg twice daily or 1200 mg once daily for 14 days to patients with newly diagnosed drug-sensitive pulmonary tuberculosis, to assess its safety, tolerability, pharmacokinetics, and early bactericidal activity (EBA) in sputum. The main objective of the study was to determine if sutezolid treatment resulted in significant reduction in log sputum CFU counts with 5%–95% confidence intervals that excluded zero. The study also examined bactericidal activity in *ex vivo* whole blood cultures infected with *M. tuberculosis* (whole blood bactericidal activity or WBA), as well as resistance prevention in sputum (measured as changes in MICs during the treatment period).

## Methods

### Protocol

The protocol for this trial and supporting CONSORT checklist are available as supporting information; see Checklist S1 and Protocol S1.

### Subjects

All subjects provided written informed consent according to ICH guidelines. Subjects consisted of men and women aged 18–65 years, with chest radiographs consistent with pulmonary tuberculosis, positive sputum acid-fast smears, culture or molecular confirmation of drug-susceptible *M. tuberculosis*, random blood glucose <150 mg/dL, hemoglobin >8 g/dL, serum creatinine <2 mg/dL, AST <3x ULN, total bilirubin <1.3 mg/dL. Serologic testing was not performed for hepatitis B or C, nor were these exclusion criteria. Subjects were either HIV-1 uninfected, or HIV-1 infected with CD4 T cell counts >350/mm^3^ and not currently receiving anti-retroviral therapy. Patients receiving monoamine oxidase (MAO) inhibitors, tricyclic antidepressants, or adrenergic agonists such as pseudoephedrine or phenylpropanolamine within the preceding 7 days were excluded (due to potential concerns regarding MAO-A inhibition), as were patients who had received drugs with anti-tuberculosis activity within the preceding 6 months, had a positive test for urinary isoniazid metabolite at the time of screening, or who had significant hemoptysis.

### Treatments

After providing written informed consent, subjects were randomly assigned in blocks of 7–9 to sutezolid 600 mg BID (N = 25) or 1200 mg QD (N = 25), or to a positive control of weight adjusted fixed dose combination tablets (Rifafour© e275; Sanofi-Aventis, Midrand, South Africa) consisting of isoniazid, rifampin, pyrazinamide, and ethambutol (HRZE, N = 9). The purpose of this group was to ascertain comparability of laboratory methods to other EBA trials. Sutezolid was administered as 200 mg tablets. All treatments were administered on an inpatient basis. Neither subjects nor investigators were blinded to assigned treatment. Laboratory staff performing the sputum and whole blood assays was unaware of treatment allocation. After discharge all subjects were referred for a full course of standard antituberculosis treatment.

### Evaluations

Symptoms and physical exam were monitored daily. Electrocardiograms were obtained at baseline, day 1, and day 14. Routine blood and urine safety tests were monitored on days 1, 14, and 42. Pooled sputum samples were collected for 16 hrs on days −1, 0, 1, 2, 3, 4, 6, 8, 10, 12, and 14, beginning in the afternoon of the specified study day and continuing through the next morning. Days −1 and 0 were considered baseline days for statistical calculations. Treatment began in the morning of day 1, after collection of the day 0 sputum sample was completed. Sputum specimens were stored on ice at the bedside during collection and transported to the central laboratory (Department of Biomedical Sciences, Stellenbosch University, Tygerberg, South Africa) at 4°C the next morning. Pooled specimens were homogenized by stirring for 30 minutes, then digested with an equal volume of Sputasol (Oxoid, Cambridge UK) at a final dithiothreitol concentration of 10%. An aliquot was removed and a series of 10-fold dilutions prepared in saline with Tween 80 for log CFU determination on 7H11 agar with Selectatab (polymyxin B, ticarcillin, amphotericin B, trimethoprim, MAST, Bootle, Merseyside, UK) added. Log CFU determinations were performed on specimens collected on days −1, 0, 1, 2, 4, 6, 10, and 14. A second aliquot was decontaminated with 1% NaOH-N-acetyl-L-cysteine, diluted with PBS and centrifuged at 4°C and 3000 g for 15 minutes. The supernatant was discarded and the pellet resuspended in 1.5 ml PBS. 500 µl of this was used to inoculate a Mycobacteria Growth Indicator Tube (MGIT, Becton Dickinson, Sparks, USA) supplemented with OADC (oleic acid, albumin, dextrose, catalase) and PANTA (polymyxin B, amphotericin B, nalidixic acid, trimethoprim, azlocillin). MGITs were incubated at 37°C in a BACTEC MGIT 960 instrument until flagged positive or for a maximum of 42 days in case no growth was detected. Time to positivity (TTP) in MGIT cultures was recorded for all specimens. Contamination was excluded by placing one drop of positive liquid culture on a blood agar plate (NHLS, Cape Town, South Africa) and by incubating for 48 hours at 37°C without visible growth. Contaminated cultures were excluded from analysis.

Blood was collected for WBA on prior to treatment on day 1. Blood was collected for PK and WBA 8 and 12 hrs post dose on day 13, and at 0, 1, 2, 3, and 6 hrs post dose on day 14. Plasma was separated immediately after collection and stored at −20°C for PK determinations. Total plasma concentrations of PNU-100480 and PNU-101603 were determined using a validated LC-MS/MS method by Advion BioServices (Ithaca, NY, USA), as previously described [7]. Blood for WBA was stored at room temperature with slow constant rotation until a full set of samples for a subject had been collected, at which time they were transported to the laboratory. WBA against *M. tuberculosis* H37Rv was determined as previously described [7]. Briefly, *M. tuberculosis* H37Rv was grown in MGIT and frozen in aliquots at −80C. A titration experiment determined the relationship between inoculum size and TTP, and identified the volume positive in 5.5 days. Whole blood cultures consisted of heparinized blood, an equal volume of RPMI 1640 tissue culture medium (Highveld Biological, Lyndhurst, South Africa), and mycobacteria from the specified volume of mycobacterial stock. After 72 hrs, cells were sedimented, the liquid phase removed, and blood cells disrupted by hypotonic lysis. Bacilli were recovered and inoculated into MGIT and incubated until flagged as positive. Log change in viability was calculated as log(*final*) – log(*initial*), where *final* and *initial* are the volumes corresponding to TTP of the completed cultures and its inoculum control, respectively, based on the titration curve. Results were expressed as log change per day of whole blood culture. Cumulative WBA over 24 hrs was calculated as the AUC_0-24_, and expressed as Δlog/d•d, or simply as log change.

### Minimal inhibitory concentration (MIC) testing

MICs of sutezolid and its major metabolite were determined using MGIT. Sterile stock solutions of sutezolid and PNU-101603 10 mg/ml were prepared in DMSO. Testing was performed using a series of 2-fold reductions in drug concentrations from 4.0 to 0.062 µg/ml. Growth in drug-containing tubes was compared to that of a positive growth control in which the inoculum was diluted 1∶100 in saline. The MIC was defined as the lowest concentration of drug without growth (GU<100) at the time when growth was detected in the positive control.

### Safety reporting

Specific guidance was provided to investigators for the identification of serious adverse events (SAEs), which were defined as those resulting in death, being life-threatening, requiring or prolonging hospitalization, resulting in persistent or significant disability or incapacity, or resulting in congenital anomaly. Guidance was provided for classification of abnormal test results as adverse events and as well as criteria for laboratory abnormalities of potential clinical concern. Potential cases of drug-induced liver injury were defined by Hy's criteria [8]. AE severity was clinically assessed as mild, moderate, or severe according to the extent to which it interfered with the subject's usual function (none, partially, or significantly). The accuracy of all entered study data was verified by independent study monitors.

### Statistics

The sample size for the sutezolid arms was selected to have 80% power to detect a change from baseline in sputum log_10_ CFU/mL from baseline to day 2 of −0.18 (equal to that observed for linezolid 600 mg QD), using a one-sided test at the 0.05 significance level, and assuming between subject variability (SD) of 0.35 [9], [10]. Sputum log_10_ CFU/ml was analyzed by mixed effects model repeated measures analysis (MMRM), using fixed effects for treatment, baseline values, study day as a categorical variable, and study day by treatment interaction, and a random effect for subject with unstructured within subject covariances (unstructured to allow for its full estimation from present data). Rates of change from baseline to day 2, from day 2 to 14, and from baseline to day 14 were examined. Additional analyses of sputum log_10_ CFU/ml were conducted using ANCOVA of change from beginning to end of each period, using ANCOVA of changes based on regression slope, and using mixed effects model repeated measures analysis with day as a regression variable. 90% confidence limits (5%–95%) were calculated to be consistent with a one-sided test at the 0.05 significance level. Changes in MGIT TTP were analyzed similarly. Cumulative WBA was analyzed by an ANCOVA with fixed effects for treatment and baseline, and a random effect for subject. MIC results were analyzed post-treatment vs. pre-treatment post hoc, separately for parent/metabolite and BID/QD schedules due to different characteristics and data distributions, by generalized linear mixed model repeated measures analysis (GLMMRM), using multinomial cumulative logit link with proportional odds. GLMMRM is potentially a more sensitive test in the case of missing data. A nonparametric sign test was also performed post hoc on MIC results as a sensitivity analysis, as a more conservative method. Two-sided testing was used on these post hoc analyses. There was no multiplicity adjustment for tests in different datasets.

### Ethical review

This study received ethical approval from the University of Cape Town Faculty of Health Sciences Human Research Ethics Committee, Cape Town, South Africa, and from Pharma-Ethics, Lyttelton Manor, South Africa. The study received regulatory approval in South Africa and was registered on clinicaltrials.gov as NCT01225640.

### Role of the funding source

This study was funded by Pfizer, Inc. Pfizer employees contributed to study design, data analysis, and data interpretation. All authors had full access to all study data and had final responsibility for the decision to submit for publication. Rights to sutezolid were acquired by Sequella, Inc. in 2013.

## Results

### Subjects

**Figure 1:**
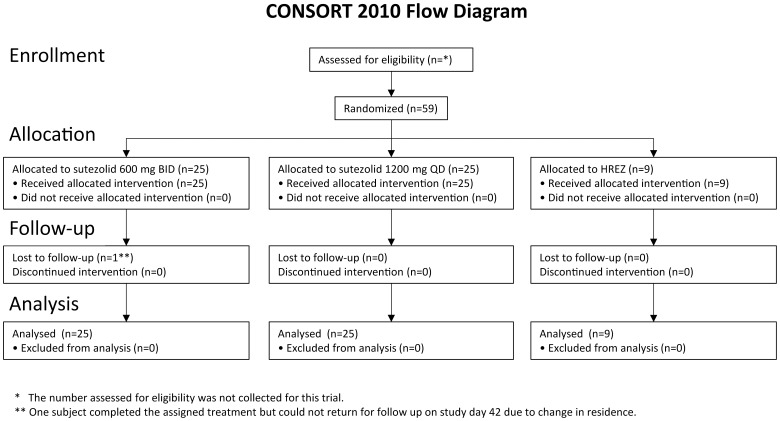
CONSORT flow diagram of study enrollment.

Enrollment began at both sites in August 2011; study participation was completed by December 2011. A CONSORT flow diagram showing enrollment is shown in figure 1. Subject demographic and baseline characteristics were similar among the 3 arms (table 1). Subjects were primarily male (80%) and <45 years of age (81%). Four subjects were HIV-1 seropositive, with CD4 T cell counts ranging from 643 to 812 cells/µl. The baseline sputum bacillary burden was approximately 10^7^ CFU/ml, similar to other EBA trials. All subjects completed their assigned treatments, began subsequent standard TB treatment without interruption, and were included in the full analysis set.

**Table 1:** Baseline subject characteristics by treatment arm.

	Sutezolid	Sutezolid	RHZE
	600 mg BID	1200 mg QD	
Number of subjects	25	25	9
Age (years, mean ± SD)	32.3±9.0	34.1±11.7	33.8±11.8
Sex (male/female)	20/5	20/5	7/2
Race (Black/Other)	11/14	8/17	3/6
Weight (kg, mean ± SD)	54.6±6.5	51.1±6.7	51.3±7.5
Height (cm, mean ± SD)	167.4±8.2	167.0±6.1	166.5±11.8
BMI (kg/m^2^, mean ± SD)	19.6±2.9	18.3±1.8	18.4±0.5
Baseline microbiology:			
log CFU/ml (mean ± SD)	6.88±1.11*	6.92±1.20	7.22±0.71
TTP (hours, mean ± SD)	125.0±42.5	106.8±34.8	115.4±34.1
WBA (Δlog/d, mean ± SD)	0.176±.126^†^	0.221±.080	0.190±.026^§^

*N = 23; ^†^N = 21; ^§^N = 5. BMI = body mass index; TTP = time to positivity in automated liquid culture; WBA = whole blood bactericidal activity.

### Sputum bactericidal activity

**Figure 2:**
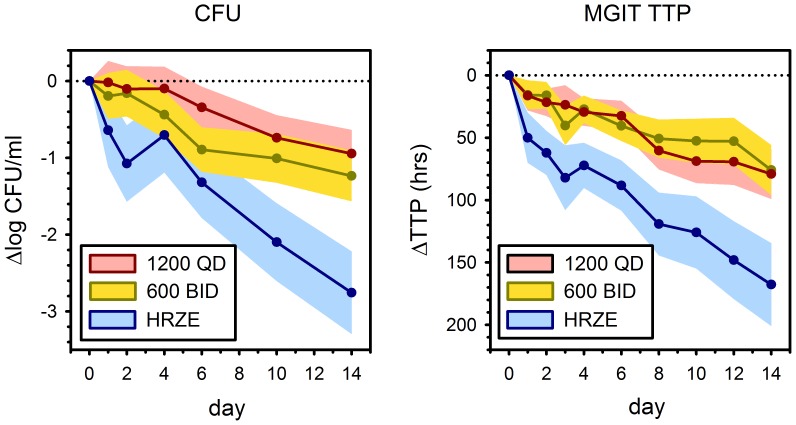
Bactericidal activity in sputum according to treatment arm as assessed by colony counts (left) and time to positivity in automated liquid culture (MGIT TTP, right). Lines indicate prediction and shading 90% confidence interval (CI) as determined by mixed effects model repeated measures analysis, using day as a categorical variable. The vertical axis of the right hand figure is inverted to facilitate visual comparison with CFU findings. At 14 days, the 90% CI of all treatments excluded zero.

Both dosing schedules of sutezolid resulted in changes in sputum log_10_ CFU over the entire period of treatment that excluded zero (table 2 and figure 2 left panel). Separate analysis of days 0–2 and 2–14 showed the effect only was significant during the later interval. A trend toward a superior effect was apparent in mean values across days when sutezolid was given as 600 mg BID (figure 2), but the confidence intervals for the two dosing schedules largely overlapped (figure 2 and table 2). Effects in HRZE-treated subjects were similar to those reported in other EBA trials [11].

**Table 2:** Rate of change of sputum log_10_ CFU counts per day according to mixed effects modeling repeated measures analysis, using fixed effects for treatment, day, treatment by day interaction, and baseline values (defined as the mean of days −1 and 0).

Treatment	Estimate	SE	90% CI	P*
**Baseline to day 2**				
Sutezolid 600 mg BID	−0.080	0.091	−0.232 to 0.072	0.192
Sutezolid 1200 mg QD	−0.051	0.088	−0.199 to 0.096	0.281
RHZE	−0.537	0.148	−0.785 to −0.289	0.0003
**Day 2 to 14**				
Sutezolid 600 mg BID	−0.090	0.018	−0.120 to −0.059	<.0001
Sutezolid 1200 mg QD	−0.070	0.017	−0.099 to −0.041	<.0001
RHZE	−0.140	0.030	−0.190 to −0.090	<.0001
**Baseline to day 14**				
Sutezolid 600 mg BID	−0.088	0.014	−0.112 to −0.065	<.0001
Sutezolid 1200 mg QD	−0.068	0.013	−0.090 to −0.045	<.0001
RHZE	−0.197	0.023	−0.235 to −0.158	<.0001

*P values indicate the likelihood of no difference from zero as determined by a one-tailed test.

Changes in MGIT TTP analyzed by MMRM are illustrated in the right panel of figure 2. Like CFU counts, the 90% CI of the effects on TTP of both dosing schedules excluded zero. However, unlike CFU counts, no lag period during early treatment was apparent (*i.e*., changes from baseline to day 2 were statistically significant), nor was any trend apparent toward superiority of 600 mg BID dosing.

### Whole blood bactericidal activity

**Figure 3:**
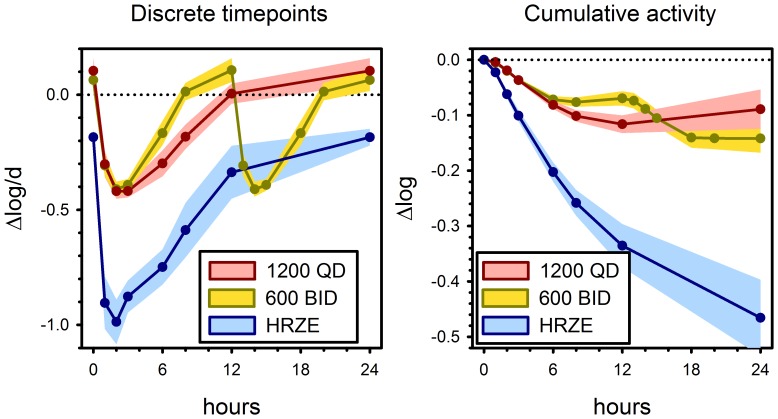
Bactericidal activity against M. tuberculosis H37Rv in whole blood culture (WBA) according to treatment arm, at discrete time points (left), and as cumulative effect (right). Lines and shading indicate means and 90% confidence intervals (CI).

Prior to the start of treatment, there was mean growth of *M. tuberculosis* H37Rv of approximately +0.2 log/d in *ex vivo* whole blood cultures (table 1), corresponding to a doubling time of approximately 36 hrs. Sutezolid treatment resulted in readily detectable bactericidal activity whether administered as a single or divided dose (figure 3). Both doses produced maximal killing of approximately −0.4 log/d, occurring 2–3 hrs post dose. Analysis of cumulative activity over 24 hr by ANCOVA indicated log_10_ estimates of -0.142±0.020 and −.089±0.020 (mean ± SE) for BID and QD dosing, respectively. Although the 90% CI of difference between the 2 schedules excluded zero (−0.100 to −0.005), the difference only approached significance at the two-tailed 5% level (P = 0.068). There was no correlation between cumulative WBA and EBA from baseline to day 2 or from days 2–14 among sutezolid-treated patients (all R<0.2, P>0.2).

### Minimum inhibitory concentrations

**Figure 4:**
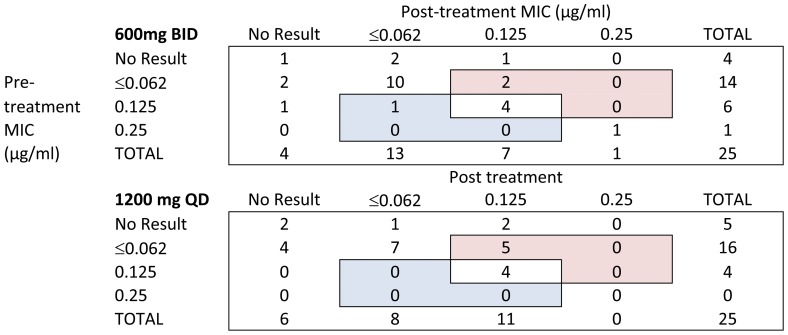
Distributions of minimal inhibitory concentrations (MICs) to sutezolid (PNU-100480) pre and post treatment (rows and columns, respectively), according to dosing schedule. Values in each cell indicate numbers of patients. Cells shaded red are those in which MIC values increased, whereas they decreased in those shaded blue. No change was apparent in MIC values of the metabolite (PNU-101603, not shown).

MIC testing of sutezolid and its major metabolite was incomplete, lacking 38/200 (19%) results due to time and resource constraints. Median MIC values for the parent and metabolite at baseline were ≤0.062 µg/ml and 0.500 µg/ml, respectively, in both treatment arms. Testing was repeated on day 15 to determine if values increased due to treatment. In patients treated with sutezolid 1200 mg QD, the median MIC of the parent on day 15 was 0.125 µg/ml. This reflected 5 instances in which MICs increased *vs*. none in which they decreased (figure 4). However, the significance level of this change (two-tailed GLMMRM: *P* = .034; two-tailed sign test: *P* = .063) does not account for the 4 comparisons conducted for the 4 different datasets (2 treatment arms, parent and metabolite) in post-hoc testing. All other median MICs were unchanged (*P*≥.727).

### Plasma pharmacokinetics

**Figure 5:**
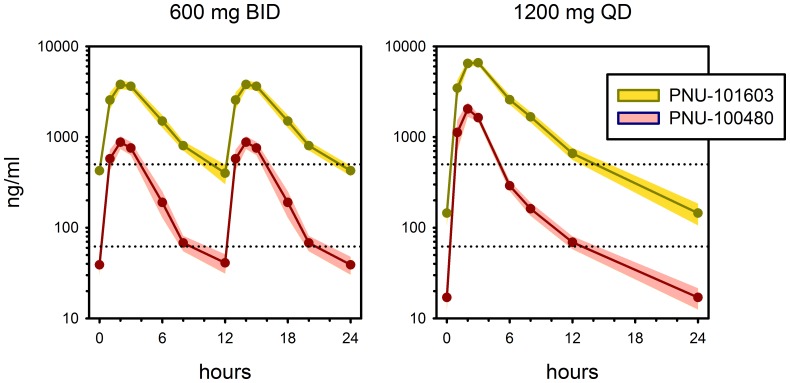
Plasma concentrations of sutezolid (pink) and its major metabolite (yellow) at steady state (day 13–14) in patients treated with sutezolid 600 mg BID (left) or 1200 mg QD (right). Solid lines indicate medians; shading indicates 90% CI. Lower and upper dotted lines indicate median pre-treatment MIC values for parent and metabolite, respectively.

Plasma concentrations of sutezolid and its major metabolite (PNU-101603) on day 14 are shown in figure 5. Key PK parameters are summarized in table 3. While the AUC_0__−24_ values were comparable between the QD and BID dosing regimens for both sutezolid and PNU-101603, administration of sutezolid as a single daily dose resulted in a doubling of the Cmax but slightly less than a doubling of that of the major metabolite. When dosed at 600 mg BID, median plasma concentrations of parent and major metabolite remained above their respective median MIC values for 71% and 89% of the dosing interval. In contrast, dosing at 1200 mg QD resulted in median supra-MIC plasma concentrations for 53% and 57% of the dosing interval, respectively. The observed mean AUC_0–24_ of parent and metabolite appeared to range from 63–71% and 86–91%, respectively, of values observed in phase 1, whereas C_max_ values of parent and metabolite equaled those observed in healthy volunteers in phase 1 [5].

**Table 3:** Geometric mean pharmacokinetic parameters in patients of sutezolid and its major metabolite following dosing for 14 Days.

Dose	N	sutezolid (PNU-100480)		major metabolite (PNU-101603)	
		Cmax	AUC_0-24_	Cmax	AUC_0–24_
		*ng/mL (CV)*	*ng•h/mL (CV)*	*ng/mL (CV)*	*ng•h/mL (CV)*
600 mg BID	25	986 (36%)	6494 (35%)	4355 (20%)	39140 (18%)
1200 mg QD	25	1972 (50%)	7127 (36%)	7050 (18%)	36820 (22%)

CV = coefficient of variation.

### Safety

**Figure 6:**
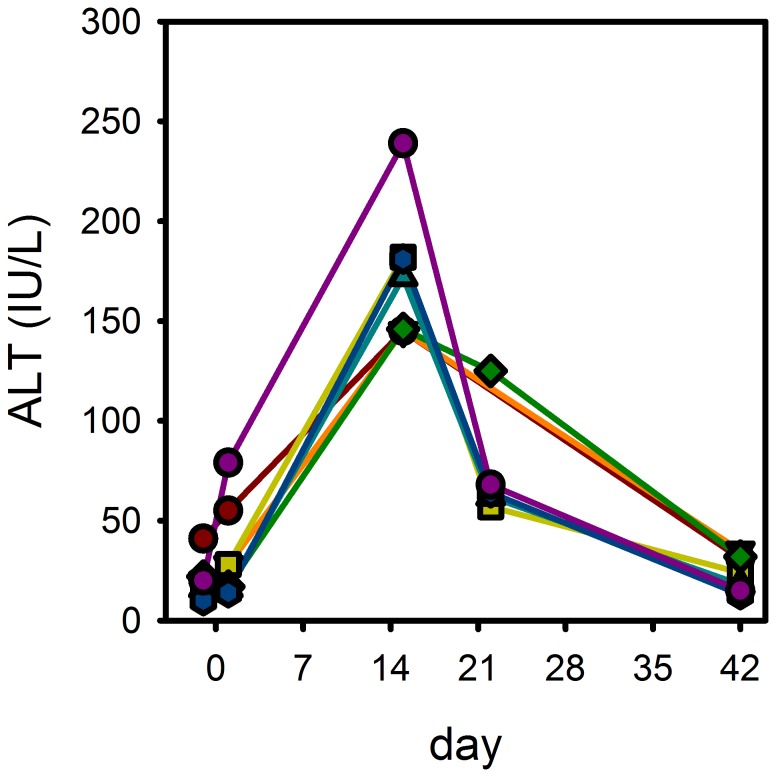
Treatment-emergent ALT increases in sutezolid-treated subjects.

Treatment with sutezolid was generally safe and well tolerated. No subject required dose reduction or premature discontinuation due to adverse events or abnormal laboratory parameters. There were no instances of anemia or thrombocytopenia. There was no effect of sutezolid on the QTc interval, with changes from baseline to day 14 of −4.2±14.5 msec and −3.1±12.1 msec (mean±SD) in the 600 mg BID and 1200 mg QD arms, respectively. Treatment-emergent adverse events were distributed evenly across the sutezolid arms and were mainly classified as mild (n = 26) or moderate (n = 13) in severity. A total of 7 sutezolid-treated subjects (14%) experienced mild or moderate increases in alanine transaminase (ALT). Cases were distributed in both men and women (5∶2) and in both the BID and QD treatment arms (4∶3). None occurred in HIV-1 seropositive individuals. No other predisposing factors were identified. ALT values for these subjects increased from 34±24 IU/L at baseline (mean±SD) to 173±34 IU/L on day 15 (reference range, 6–48 IU/L) (figure 6). ALT increases were accompanied by smaller increases in AST, but not by changes in alkaline phosphatase or bilirubin. None of the subjects experienced symptoms of drug-induced liver injury. None met Hy's criteria for serious liver injury [8]. All 7 subjects completed their assigned sutezolid treatment and began standard TB therapy without interruption. Five of the 7 subjects were recalled for repeat testing on day 22, by which time values had declined to 75±28 IU/L. Values for all patients had returned to normal on day 42 (24±9 IU/L).

One adverse event met study criteria for both serious and severe: hemoptysis occurred in 1 subject 14 days after the last dose of sutezolid 600 mg BID. It was thought by both the investigator and sponsor to be related to tuberculosis rather than its treatment. Lastly, one instance of grade 1 peripheral neuropathy occurred in a patient assigned to sutezolid 600 mg BID. However, its onset occurred after sutezolid treatment had been completed, and was attributed by the investigator to the standard TB treatment that followed. A listing of all treatment-emergent adverse events of all potential causes can be found in table S1.

## Discussion

This is the first study of sutezolid in patients with pulmonary tuberculosis. The main findings were that doses of 600 mg BID and 1200 mg QD given for 14 days were generally safe, well tolerated, and resulted in readily detectable bactericidal activity in both sputum and blood. Its effects in sputum were sustained throughout the full period of treatment. These findings support further development of sutezolid as a component of new tuberculosis regimens.

Prior studies have separately examined the mycobactericidal activity of linezolid in sputum and blood. One study in a total of 19 TB patients found linezolid 600 mg BID and 600 mg QD produced effects approaching statistical significance of −0.26 and −0.18 log_10_ CFU/ml/day, respectively, during the first 2 days of treatment, but not subsequently [9]. In contrast, the present study found no significant effect on sputum CFU counts during the first 2 days of treatment, but did find a significant, sustained effect subsequently. Effects during early TB treatment are thought to represent activity against extracellular, metabolically active bacilli in cavities. This bacillary subpopulation is critical for TB transmission and for the emergence of resistance, but it appears not to be involved in persistence and relapse. The sustained activity of sutezolid observed in the present trial may indicate enhanced ability to sterilize tissues and thereby shorten the required duration of treatment, as has been shown in the mouse model [4]. However, there is no evidence at present to indicate a relationship between EBA during the first 2 weeks of TB treatment and the total duration required for relapse-free cure [12].

This trial is the first in which intracellular bactericidal activity in blood of TB patients has been assessed in parallel with that in sputum. Sutezolid and its main metabolite appear to act against distinct mycobacterial subpopulations. *In vitro*, extracellular killing is mainly due to the metabolite, whereas killing of intracellular mycobacteria, such as in the whole blood model, is mainly due to the parent [13], [14]. Sutezolid shows superior activity *vs*. linezolid in the whole blood model [5]. In one study, whole blood bactericidal activity during TB treatment correlated with 2 month sputum culture status [15], which in turn is a predictor of relapse [16]. Longer trials will be required to better assess the sterilizing activity of sutezolid and to determine the required duration of new sutezolid-containing regimens. Future trials may also consider studying each patient using his or her own isolate in whole blood culture to enhance its predictive value [15].

The mycobactericidal activity of sutezolid in the whole blood and hollow fiber models is mainly dependent on time rather than concentration [5], [13]. Given this observation, the relatively short plasma half-lives of both the parent and metabolite (approximately 4 hrs) ordinarily would favor divided rather than single daily dosing. In the present study, divided dosing tended to show superior bactericidal activity in blood, although this at best was only at the threshold of statistical significance. This trial was neither intended nor powered to detect a difference between single and divided daily dosing. Longer clinical trials will be required to determine the potential clinical impact of single *vs*. divided daily dosing when sutezolid is combined with other TB drugs in novel regimens.

Prevention of resistance is an essential characteristic of TB drugs. Experience with linezolid indicates this characteristic may be distinct from EBA, as linezolid lacks sustained EBA [9], yet shows remarkable ability to prevent acquired resistance even when added as a single new drug to a failing regimen [3]. No conclusion can be drawn regarding the MIC findings in this trial due to the post-hoc nature of the analysis and the relatively small magnitude of observed increases. However, EBA trials are intended to closely study a small number of subjects during a short period of treatment so as to inform the design of future trials. Studies of sutezolid in the hollow fiber model have reported similarly reduced bactericidal activity and earlier emergence of resistance with simulated once daily dosing [6]. Pre- and post-treatment MIC testing should be considered to assess resistance prevention in future EBA trials.

Peak concentrations of sutezolid and its main metabolite in TB patients in this study equaled those observed in previous studies in healthy volunteers, whereas total exposure to the parent was reduced by one-quarter. This decreased exposure appears to be the cause of the reduced bactericidal activity in whole blood cultures at later time points in patients as compared to those previously reported in healthy volunteers [5]. Macrophage production of oxygen radicals is increased in TB as part of the antimycobacterial host response. Non-enzymatic oxidation may contribute to sutezolid metabolism. Further studies are warranted to determine whether the rate of metabolic clearance of sutezolid slows as inflammation resolves during TB treatment, as this may restore intracellular bactericidal activity to levels observed in volunteers.

Mild to moderate increases in hepatic alanine aminotransferase were observed in 14% of sutezolid-treated subjects in this study. In contrast, ALT levels had remained within the normal range in all subjects during phase 1 studies of sutezolid in healthy volunteers. Early, mild, transient, asymptomatic increases in liver enzymes are common in patients treated with anti-TB drugs [17]. There is increasing evidence that these represent heightened susceptibility to oxidative liver injury due to depletion of glutathione, which ordinarily protects against such damage. Intracellular stores of glutathione are depleted, and the ratio of reduced to oxidized glutathione diminished, following experimental *M. tuberculosis* infection of guinea pigs [18]. Glutathione levels are low in TB patients, with the lowest levels occurring in those patients who go on to experience TB drug-induced liver injury [19]–[21]. Cysteine availability is the limiting step in glutathione synthesis. A small prospective randomized trial reported that TB drug-induced liver injury could be prevented by supplementation with N-acetylcysteine (NAC) [22]. A study of NAC may be considered to reduce overall risk in future trials of new TB drugs, including sutezolid.

In summary, this first trial of the oxazolidinone sutezolid in patients with pulmonary tuberculosis found the drug to be generally safe, well tolerated, and with readily detectable bactericidal activity in sputum and blood. Further studies of this promising compound are warranted.

## Supporting Information

Table S1 **Adverse events (all causation) according to severity and treatment arm.**
(DOCX)

Checklist S1 **CONSORT Checklist.**
(DOC)

Protocol S1 **Trial Protocol.**
(PDF)
