# Supplementary material for: Mycobactericidal Activity of Sutezolid (PNU-100480) in Sputum (EBA) and Blood (WBA) of Patients with Pulmonary Tuberculosis
Source: PLoS One. 2014 Apr 14;9(4):e94462. doi: 10.1371/journal.pone.0094462 (PMC3986205; doi:10.1371/journal.pone.0094462)
Supplement: Table S1 — Adverse events (all causation) according to severity and treatment arm. (DOCX) [file pone.0094462.s001.docx]

**Supplemental materials**

**Table S1.** Adverse events (all causation) according to severity and treatment arm.

|  | **Sutezolid 600mg BID (n=25)** | | | **Sutezolid 1200mg QD (n=25)** | | | **RHZE (n=9)** | | |
| --- | --- | --- | --- | --- | --- | --- | --- | --- | --- |
|  | Mild | Mod | Sev | Mild | Mod | Sev | Mild | Mod | Sev |
| CARDIAC |  |  |  |  |  |  |  |  |  |
| Tachycardia | 0 | 0 | 0 | 1 | 0 | 0 | 0 | 0 | 0 |
| GASTROINTESTINAL |  |  |  |  |  |  |  |  |  |
| Abdominal pain | 0 | 0 | 0 | 0 | 0 | 0 | 1 | 0 | 0 |
| Constipation | 1^†^ | 0 | 0 | 0 | 0 | 0 | 0 | 0 | 0 |
| Nausea | 0 | 0 | 0 | 0 | 0 | 0 | 2 | 0 | 0 |
| Rectal haemorrhage | 0 | 1^†^ | 0 | 0 | 0 | 0 | 0 | 0 | 0 |
| Vomiting | 1 | 0 | 0 | 0 | 0 | 0 | 0 | 0 | 0 |
| GENERAL |  |  |  |  |  |  |  |  |  |
| Chest pain | 0 | 0 | 0 | 0 | 1 | 0 | 0 | 0 | 0 |
| Feeling hot | 0 | 0 | 0 | 0 | 0 | 0 | 1 | 0 | 0 |
| Oedema peripheral | 0 | 0 | 0 | 1 | 0 | 0 | 0 | 0 | 0 |
| INFECTIONS, INFESTATIONS |  |  |  |  |  |  |  |  |  |
| Gastroenteritis | 0 | 1 | 0 | 0 | 0 | 0 | 0 | 0 | 0 |
| Pelvic inflammatory dis | 0 | 0 | 0 | 0 | 0 | 0 | 1 | 0 | 0 |
| Rash pustular | 1 | 0 | 0 | 0 | 0 | 0 | 0 | 0 | 0 |
| INVESTIGATIONS |  |  |  |  |  |  |  |  |  |
| ALT increased | 0 | 4^§^ | 0 | 0 | 3^§^ | 0 | 0 | 0 | 0 |
| METABOLISM |  |  |  |  |  |  |  |  |  |
| Hyperglycaemia | 1 | 0 | 0 | 0 | 0 | 0 | 0 | 0 | 0 |
| Increased appetite | 1 | 0 | 0 | 0 | 0 | 0 | 0 | 0 | 0 |
| MUSCULOSKELETAL |  |  |  |  |  |  |  |  |  |
| Arthralgia | 1 | 0 | 0 | 0 | 0 | 0 | 1 | 0 | 0 |
| Musculoskeletal pain | 0 | 0 | 0 | 1 | 0 | 0 | 0 | 0 | 0 |
| Myalgia | 1 | 0 | 0 | 0 | 0 | 0 | 1 | 0 | 0 |
| NERVOUS SYSTEM |  |  |  |  |  |  |  |  |  |
| Headache | 0 | 0 | 0 | 1 | 0 | 0 | 0 | 0 | 0 |
| Neuropathy peripheral | 1 | 0 | 0 | 0 | 0 | 0 | 0 | 0 | 0 |
| Somnolence | 1 | 0 | 0 | 1 | 0 | 0 | 0 | 0 | 0 |
| PSYCHIATRIC |  |  |  |  |  |  |  |  |  |
| Insomnia | 0 | 0 | 0 | 1 | 1 | 0 | 0 | 0 | 0 |
| RENAL AND URINARY |  |  |  |  |  |  |  |  |  |
| Urinary retention | 0 | 0 | 0 | 0 | 0 | 0 | 0 | 1 | 0 |
| REPRODUCTIVE |  |  |  |  |  |  |  |  |  |
| Erectile dysfunction | 0 | 0 | 0 | 0 | 0 | 0 | 0 | 1 | 0 |
| Genital burning sensation | 0 | 0 | 0 | 0 | 0 | 0 | 1 | 0 | 0 |
| RESPIRATORY |  |  |  |  |  |  |  |  |  |
| Haemoptysis | 0 | 0 | 1* | 1 | 1 | 0 | 1 | 0 | 0 |
| Pleuritic pain | 0 | 0 | 0 | 2 | 0 | 0 | 0 | 0 | 0 |
| SKIN |  |  |  |  |  |  |  |  |  |
| Pruritus | 5 | 1 | 0 | 1 | 1 | 0 | 1 | 0 | 0 |
| Rash | 1 | 1 | 0 | 1 | 0 | 0 | 0 | 0 | 0 |
| TOTAL | 15 | 7^§^ | 1* | 11 | 6^§^ | 0 | 10 | 2 | 0 |

*Hemoptysis occurred on day 28, 14 days after the last PNU-100480 dose. ^†^Rectal bleeding and constipation occurred at the same time point in the same patient. ^§^Two of the 7 cases of ALT increase (1 in each sutezolid arm) were not designated as adverse events by the investigator.
