# Supplementary material for: Mycobactericidal Activity of Sutezolid (PNU-100480) in Sputum (EBA) and Blood (WBA) of Patients with Pulmonary Tuberculosis
Source: PLoS One. 2014 Apr 14;9(4):e94462. doi: 10.1371/journal.pone.0094462 (PMC3986205; doi:10.1371/journal.pone.0094462)
Supplement: Protocol S1 — Trial Protocol. (PDF) [file pone.0094462.s003.pdf]

CLINICAL PHARMACOLOGY PROTOCOL

A PHASE 1 STUDY TO EVALUATE THE SAFETY, TOLERABILITY, AND PHARMACOKINETICS OF PNU-100480 (PF-02341272) AFTER FIRST TIME ADMINISTRATION OF ASCENDING ORAL DOSES TO HEALTHY ADULT SUBJECTS UNDER FED AND FASTED CONDITIONS

**Compound:**	PNU-100480 (PF-02341272)
**Compound Name (if applicable):**	N/A
**US IND Number (if applicable):**	104, 806
**Protocol Number:**	B1171001
**Phase:**	Phase 1
**Version and Date:**	Final Protocol Amendment #1<br>06 May 2009<br><br>Original Protocol<br>12 March 2009

This document contains confidential information belonging to Pfizer. Except as otherwise agreed to in writing, by accepting or reviewing this document, you agree to hold this information in confidence and not copy or disclose it to others (except where required by applicable law) or use it for unauthorized purposes. In the event of any actual or suspected breach of this obligation, Pfizer must be promptly notified.

090177e180ac49ac\1.0\Approved\07-May-2009 14:41

SCHEDULE OF ACTIVITIES

**Protocol Activity**	Screen	Day 0	Day 1									Day 2		Day 3		Day 4[j]	Follow Up (7-14 days postdose)
**Hours Post Dose**			0 (Predose)	1	2		3	4	6	8	12	16	24	36	48	72	
Informed Consent	X																
Admission to Clinical Research Unit		X															
Medical History[a]	X	X															
Physical Examination[b]	X	X													X[c, i]	X[c]	X
Height and Weight	X																
Single Supine BP/PR	X		X	X	X		X	X		X	X		X	X	X	X	X
Hematology	X	X											X		X[i]	X	X
Chemistry	X	X											X		X[i]	X	X
Urinalysis	X	X											X		X[i]	X	X
Urine Cotinine[d]	X	X															
Urine Drug Screen	X	X															
Serum FSH[e]	X																
Triplicate 12-Lead ECG			X	X	X		X	X		X	X		X	X	X[i]	X	
Single 12-Lead ECG	X																X
Telemetry[f]			X	X	X		X	X		X							
Study Treatment Administration			X														
PK Blood Sampling			X	X	X		X	X		X	X	X	X	X	X	X[k]	
Adverse Event Monitoring			X	X	X			X		X	X	X	X	X	X	X	X
Review Prior/Concomitant Medication[g]	X	X															X
Whole Blood Bactericidal Activity Sampling[h]			X		X		X		X		X		X				
Discharge from CRU															X[i]	X	

[a] Including drug, alcohol and tobacco use.

[b] Full physical exam can be performed at Screening or on Day 0 (Period 1 only), at the discretion of the CRU staff. Limited physical exams will be performed at all other timepoints. At follow-up, a physical exam will only be performed if an AE is unresolved, or at the discretion of the investigator.

[c] Limited physical exam at Period 3 only for Cohort 1 and Period 4 only for Cohort 2.

[d] Urine cotinine will be done at Screening and Day 0 in Period 1 only. Subjects will be instructed to refrain from smoking for the duration of the study.

[e] Serum FSH for women 45-55 years of age (Screening only) to confirm non-childbearing potential status, if applicable.

090177e180ac49ac\1.0\Approved\07-May-2009 14:41

[f] Telemetry will be performed from approximately 2 hours predose (Period 1 only) through 8 hours postdose.

[g] Prior medication includes period prior to dosing; concomitant medication not allowed during the study, except acetaminophen, as defined in protocol.

[h] Whole blood for bactericidal assay (WBA) to be collected in Cohort 1, Period 3 and Cohort 2, Periods 1, 2 and 3 only.

[i] When not collecting a 72 hour pharmacokinetic analysis sample.

[j] If subject is not discharged on Day 3.

[k] PK sample to be collected for Cohort 1, Period 2 and 3, Cohort 2, Period 1, 2, 3, 4

090177e180ac49ac\1.0\Approved\07-May-2009 14:41

TABLE OF CONTENTS

1. INTRODUCTION 9
1.1. Indication 10
1.2. Background and Rationale 10
1.2.1. Preclinical Toxicology Findings 11
1.2.1.1. 6-Week Pivotal Rat Study 12
1.2.1.2. 6-Week Pivotal Dog Study 13
1.2.1.3. Relationship of Findings to Pharmacokinetics 13
1.2.1.4. Target Organ Toxicity 14
1.2.1.4.1. Hematopoietic System 15
1.2.1.4.2. Reproductive Organs 15
1.2.1.4.3. Lung 16
1.2.1.4.4. Gastrointestinal System 16
1.2.1.4.5. Liver 17
1.2.1.4.6. Other Tissues 17
1.2.1.5. Genotoxicity 18
1.2.2. Pharmacokinetic Summary 18
1.2.2.1. Pharmacokinetics/Pharmacodynamics 20
1.2.2.2. Toxicokinetics 21
2. STUDY OBJECTIVES AND ENDPOINTS 21
2.1. Objectives 21
2.2. Endpoints 21
3. STUDY DESIGN 22
3.1. Study Overview 22
3.2. Human Pharmacokinetic Projections 23
3.3. Dose Rationale and Stopping Rules 23
4. SUBJECT SELECTION 26
4.1. Inclusion Criteria 26
4.2. Exclusion Criteria 26
4.3. Randomization Criteria 28
4.4. Life Style Guidelines 28
4.4.1. Meals and Dietary Restrictions 28
4.4.2. Alcohol, Caffeine and Tobacco 29

090177e180ac49ac\1.0\Approved\07-May-2009 14:41

4.4.3. Activity....29
4.4.4. Contraception....29
4.4.4.1. Females – Non-childbearing Potential....29
4.4.4.2. Males....30
5. STUDY TREATMENTS....30
5.1. Allocation to Treatment....30
5.2. Breaking the Blind....31
5.3. Drug Supplies....31
5.3.1. Formulation and Packaging....31
5.3.2. Preparation and Dispensing....32
5.3.3. Administration....32
5.3.4. Compliance....32
5.4. Drug Storage and Drug Accountability....32
5.5. Concomitant Medication(s)....33
6. STUDY PROCEDURES....33
6.1. Screening....33
6.2. Study Period....34
6.2.1. Day 0....34
6.2.2. Day 1....35
6.2.3. Day 2....36
6.2.4. Day 3....36
6.2.5. Day 4....37
6.3. Follow-up Visit....38
6.4. Post-Study Subject Interview....38
6.5. Subject Withdrawal....38
7. ASSESSMENTS....39
7.1. Blood Volume....39
7.2. Safety....40
7.2.1. Laboratory....40
7.2.2. Blood Pressure and Pulse Rate....41
7.2.3. Electrocardiogram (ECG)....41
7.2.4. Continuous Cardiac Monitoring by Telemetry....42

090177e180ac49ac\1.0\Approved\07-May-2009 14:41

7.3. Pharmacokinetics....42
7.3.1. Plasma for Analysis of PNU-100480 and Metabolites....42
7.3.2. Shipment of Pharmacokinetic Samples....43
7.4. Pharmacodynamics (Whole Blood Bactericidal Activity)....43
7.4.1. Whole Blood for Bactericidal Activity Sample Collection....43
7.4.2. Bactericidal Activity Sample Shipment....43
7.4.3. Analysis of Whole Blood for Bactericidal Activity....43
8. ADVERSE EVENT REPORTING....44
8.1. Adverse Events....44
8.2. Reporting Period....44
8.3. Definition of an Adverse Event....44
8.4. Abnormal Test Findings....45
8.5. Serious Adverse Events....45
8.6. Hospitalization....46
8.7. Severity Assessment....47
8.8. Causality Assessment....47
8.9. Exposure In Utero....48
8.10. Withdrawal Due to Adverse Events (See Also Section on Subject Withdrawal)....49
8.11. Eliciting Adverse Event Information....49
8.12. Reporting Requirements....49
8.12.1. Serious Adverse Event Reporting Requirements....49
8.12.2. Non-Serious Adverse Event Reporting Requirements....50
8.12.3. Sponsor Reporting Requirements to Regulatory Authorities....50
9. DATA ANALYSIS/STATISTICAL METHODS....50
9.1. Sample Size Determination....50
9.2. Efficacy Analysis....51
9.3. Pharmacokinetic Analysis....51
9.3.1. Analysis Populations....51
9.3.2. Derivation of Pharmacokinetic Parameters....51
9.3.3. Statistical Methods....52
9.4. Pharmacodynamic Analysis....53
9.4.1. Whole Blood Bactericidal Activity (WBA)....53

090177e180ac49ac\1.0\Approved\07-May-2009 14:41

9.5. Safety Analysis 53
9.5.1. Electrocardiogram (ECG) Analysis 54
9.5.1.1. Derivation of Electrocardiogram Parameters 54
9.5.1.2. Statistical Methods....................54
9.5.2. Telemetry Analysis 55
10. QUALITY CONTROL AND QUALITY ASSURANCE....................55
11. DATA HANDLING AND RECORD KEEPING 55
11.1. Case Report Forms/Electronic Data Record....................55
11.2. Record Retention 56
12. ETHICS....................56
12.1. Institutional Review Board (IRB)/Independent Ethics Committee (IEC)....................56
12.2. Ethical Conduct of the Study....................56
12.3. Subject Information and Consent 57
12.4. Reporting of Safety Issues and Serious Breaches of the Protocol or ICH GCP....................57
13. DEFINITION OF END OF TRIAL....................57
14. SPONSOR DISCONTINUATION CRITERIA 58
15. PUBLICATION OF STUDY RESULTS 58
15.1. Communication of Results by Pfizer:....................58
15.2. Publications by Investigators....................59
16. REFERENCES 61

TABLES

Table 1. Combined Concentrations of PNU-100480, PNU-101603, and PNU-101244 Associated with Key Responses 14
Table 2. Genotoxicity Studies Conducted with PNU-100480....................18
Table 3. Summary of Plasma Protein Binding (% Free) and RBC Distribution (Kp) for PNU-100480, PNU-101603, and PNU-101244....................19
Table 4. Projected Steady-State Cmax, Cmin and AUC of PNU-100480, PNU-101603, and PNU-101244 in Human after 410 mg Assuming Accumulation of PNU-101603 and PNU-101244 as Observed on Days 1 and 24 in Mouse Efficacy Study....................20
Table 5. Projected Exposures of PNU-100480 (Parent Only) after Administration of Single Dose to Humans 24

090177e180ac49ac\1.0\Approved\07-May-2009 14:41

Table 6. Projected Exposures and Safety Margin of Parent (PNU-100480) and Two Metabolites (PNU-101603 and PNU-101244) Relative to Rat 100 mg/kg Dose Exposures after Administration of Single Dose of Humans 24
Table 7. Treatment Allocation 31
Table 8. Blood Volume 40
Table 9. Safety Laboratory Assessments 40
Table 10. Pharmacokinetic Parameters 52

APPENDICES

Appendix 1. Criteria for Safety Values of Potential Clinical Concern 63
Appendix 2. ECG Values of Potential Clinical Concern 64
Appendix 3. Clinical Protocol Amendment #1 65

090177e180ac49ac\1.0\Approved\07-May-2009 14:41

1. INTRODUCTION

Despite the availability of an inexpensive therapy that is reasonably effective and well tolerated, tuberculosis (TB) continues to be a major global health problem, causing an estimated 8.8 million new cases and 1.6 million deaths annually.[1] Efforts of the past decade to control TB by the consistent application of existing tools have met with only limited success, slowing its rate of increase but failing to make substantial progress toward the goal of TB elimination. These efforts have been stymied by the spread of Human Immunodeficiency Virus/Acquired Immune Deficiency Syndrome (HIV/AIDS) in TB-endemic regions, and by the global emergence of strains of *Mycobacterium tuberculosis* resistant to present TB drugs (multi- and extensively drug resistant, M/XDR-TB).[2] Two key unmet needs have been identified with regard to TB treatment: improving success rates in M/XDR TB, and shortening treatment of drug sensitive TB without increasing the risk of relapse.

The oxazolidinones are a class of antimicrobial drugs that act by blocking translation, by preventing the formation of the initiation complex. Linezolid (LZD, Zyvox®), the only marketed member of this class, has broad-spectrum activity against Gram-positive bacteria and is approved for use in complicated skin and skin structure infections and hospital-acquired pneumonia. It is also active against many mycobacterial species, including *Mycobacterium tuberculosis*, with minimum inhibitory concentrations for 50% and 90% (MIC50 and MIC90) of 0.5 and 1 µg/mL, respectively.[3] There is no known cross-resistance with current anti-TB drugs. LZD shows activity against pulmonary tuberculosis in experimental animals and in human infection.[4, 5] As a result, LZD has been used to treat recalcitrant cases of M/XDR-TB with apparent clinical benefit.[6, 7, 8, 9] However, these uncontrolled case reports also indicate that the dose and/or duration of LZD when used in TB is limited by hematologic and neurologic toxicities.[6, 8, 10] Therefore, new oxazolidinones with more potent in vivo activity against *M. tuberculosis* and lower risk of toxicity with prolonged administration are needed.

PNU-100480 (PF-2341272) is an analogue of linezolid whose activity against *M. tuberculosis* was first reported in 1996.[11] MICs of PNU-100480 against *M. tuberculosis* are 2- to 4-fold lower than LZD MICs. Studies in mice indicate substantial potential to shorten TB treatment when PNU-100480 is administered together with other TB drugs.[12] Two active metabolites (PNU-101603 and PNU-101244) may contribute to its overall effectiveness.

The purpose of this study is to evaluate the safety, tolerability, pharmacokinetics, and bactericidal activity against *M. tuberculosis* of escalating single oral doses of PNU-100480 administered as an extemporaneously prepared solution to healthy adult male and female subjects. PNU-100480 is being evaluated in humans for the first time.

090177e180ac49ac\1.0\Approved\07-May-2009 14:41

090177e180ac49ac\1.0\Approved\07-May-2009 14:41

1.1. Indication

PNU-100480 is being developed for the treatment of tuberculosis, both drug resistant and fully drug sensitive, in patients without and with HIV-1 infection.

1.2. Background and Rationale

Complete information on PNU-100480 relevant to this First in Human (FIH) study is available in the most current version of the Investigator's Brochure (IB). The IB is the Single Reference Safety Document for this study.

The candidate PNU-100480 demonstrates in vitro activity against both drug-sensitive *M. tuberculosis* and to strains resistant to other currently licensed TB drugs. This indicates potential activity against M/XDR TB. When administered in an established infection model as a single agent to mice experimentally infected with *M. tuberculosis*, PNU-100480 showed antimycobacterial activity at every dose tested. Superior activity was apparent at 50 mg/kg/day compared to 25 mg/kg/day. However, doses greater than 50 mg/kg/day did not consistently result in greater colony forming unit (CFU) reductions. Human doses of 410-490 mg are estimated to produce blood levels of PNU-100480 equivalent to 25 mg/kg in mice.

Studies in mice also indicate that multidrug regimens that include PNU-100480 show accelerated tissue sterilization and reduced risk of relapse. This indicates a potential role of PNU-100480 to shorten TB therapy. The most active regimen in this regard consisted of PNU-100480, rifampin, pyrazinamide, and moxifloxacin. In similar studies, LZD lacked this activity, indicating this may not be a property of oxazolidinones generally. The PNU-100480 dose in these studies was 100 mg/kg/day. It is not yet known whether lower daily doses of PNU-100480 will show similar sterilizing activity.

Studies in mice also indicate that PNU-101603, a sulfoxide metabolite of PNU-100480, accumulates substantially in blood after oral administration of PNU-100480, reaching levels 11 times that of the parent compound at steady state. Levels of PNU-101244, a sulfone metabolite, reached levels 1.1 times that of the parent drug. Both metabolites have inhibitory activity against *M. tuberculosis* similar to PNU-100480 when tested individually. It is not known whether the 3 moieties have additive or otherwise favorable interactions in combination. The extent to which the metabolites accumulate in human blood or phagocytic cells is not yet known.

This study includes as 1 of its objectives to determine the bactericidal activity in blood against intracellular *M. tuberculosis* after oral dosing, and to determine the contributions of the parent drug and its metabolites to the PK/PD relationship. The whole blood culture method will be used for this purpose, as the drug concentrations in the cultures reflect those in vivo at the time of phlebotomy. One study found that whole blood bactericidal activity during TB therapy correlated with the decline in sputum CFU counts and was superior in patients whose sputum cultures converted to negative after 2 months of treatment.[13] Both of these parameters are predictors of relapse risk. Two studies found that regimens for drug sensitive TB were superior to those for MDR TB when assessed by whole blood culture,

consistent with required treatment durations and outcomes associated with these regimens.[14, 15] Mycobacteria added to heparinized blood undergo rapid and essentially complete phagocytosis, and thereby become susceptible to antimycobacterial immune mechanisms.[14,16, 17] Reduced activity of certain drugs can be demonstrated in the model when mycobacterial growth is restricted by immune pressure, similar to observations in vivo.[18] Additional studies will therefore be required in which multiple doses are administered to TB patients to more fully and accurately assess drug activity in the model and in vivo.

Myelosuppression in rats and dogs was seen with linezolid, which was also observed in patients treated longer than 28 days. In contrast, administration of PNU-100480 to rats and dogs produced myelosuppression in the rat only. The relevance of this species difference to human safety is not clear. Effects on hematopoesis will be monitored closely in this study.

Inhibition of monoamine oxidase (MAO) A and B occurs with LZD, PNU-100480, and its metabolites. Although PNU-100480 is more potent against MAO-A, the major metabolite and major circulating species (PNU-101603) is less potent. In addition, PNU-100480 and the major metabolite are less potent than LZD against the MAO-B target. MAO inhibition may cause hypertension. Such episodes may be precipitated by concomitant use of vasoconstrictors, nasal decongestants, and ingestion of tyramine-rich foods such as cheese. These will be prohibited in the present study, and blood pressure will be closely monitored. MAO inhibition may also precipitate episodes of mania or hypomania in individuals with a history of bipolar illness or depression. Persons with a history of psychiatric illness will be excluded from the present study, and concomitant use of antidepressants will be prohibited.

Isoniazid and rifampin are key components of standard TB therapy. Isoniazid has weak monoamine oxidase inhibitory activity. Adverse events due to concomitant treatment with isoniazid and antidepressants have been reported, although such events appear to be extremely rare.[19] Rifampin is a potent inducer of hepatic CYP3a. CYP3a contributes to metabolism of PNU-100480. Concomitant treatment with rifampin and PNU-100480 may affect relative drug exposures, decreasing that to PNU-100480 and increasing those of its metabolites. MDR and XDR TB are by definition resistant to both isoniazid and rifampin. Neither isoniazid nor rifampin are used in such cases. Specific studies of drug-drug interactions will be required before PNU-100480 is studied with isoniazid and rifampin-containing regimens in patients with drug-sensitive tuberculosis. Isoniazid and rifampin are both excluded as concomitant medications in the present study.

1.2.1. Preclinical Toxicology Findings

A single oral dose study with PNU-100480 was conducted in the dog to evaluate tolerance and toxicokinetics. Doses up to 200 mg/kg in a vehicle of 2% polysorbate 80 in 0.5% methylcellulose (MC) were well tolerated with combined parent and metabolite (PNU-101603 and PNU-101244) exposure of 16.5 µg/mL (C_{max}) and 222 µg·h/mL ($AUC_{[0-24]}$, combined sex).

090177e180ac49ac\1.0\Approved\07-May-2009 14:41

Exploratory and definitive repeat-dose toxicity studies were conducted with PNU-100480 in rats and dogs. In a 14-day exploratory oral in vivo toleration study, male and female Sprague-Dawley rats received PNU-100480 in 5% sulfabutylether-β-cyclodextrin (SBE-CD) at 200, 500, and 1000 mg/kg/day. A dose of 1000 mg/kg/day was a lethal dose for females, with all animals either found dead during the study or euthanized moribund. Females lost weight at all doses, and decreased weight gain was observed in males at all doses. Increases in alanine aminotransferase (ALT) and aspartate aminotransferase (AST) were observed in females at 200 and 500 mg/kg/day. Combined parent and active metabolite exposures were comparable between males and females and there was no accumulation between Day 1 and Day 14. Exposure to the parent alone was 2x - 7x higher in females than males. Combined parent and active metabolite exposures achieved in this study were approximately ~1x, 3x, and 6x (combined sex, total plasma exposures) above the clinical exposure limit of 122 μg·h/mL at 200, 500, and 1000 mg/kg/day, respectively.

Three multiple dose, exploratory studies up to 21 days in duration were conducted in dogs with various formulations. Doses up to 120 mg/kg in a vehicle of 2% polysorbate 80 in 0.5% MC were well tolerated in an exploratory 14-day study in dogs. In a 21-day study, doses up to 200 mg/kg in 0.5% MC/0.1% polysorbate 80 (Days 1-7) and 0.5% MC/2% Polysorbate 80 (Days 8-21) were well tolerated. In a second 14-day study in dogs, doses up to 500 mg/kg in 20% SBE-CD were well tolerated. Systemic exposures in dog were highest with 20% SBE-CD as a vehicle, and exposures were similar at 250 and 500 mg/kg with margins of 4- and 2-fold of the clinical exposure limit (C_{max} and $AUC_{[0-24]}$), respectively.

1.2.1.1. 6-Week Pivotal Rat Study

In a 6-week definitive study, male and female Sprague-Dawley rats received PNU-100480 in 5% SBE-CD at 30, 100, and 500→300 mg/kg/day. A mid-study evaluation of clinical pathology parameters was conducted at 3 weeks. Body weight loss in females, decreased weight gain in males, and reduced food consumption in both sexes was noted at 500 mg/kg and the high dose was reduced to 300 mg/kg for both males and females after 14 and 12 days of dosing, respectively. Body weight loss in females, decreased body weight gain in males, and decreased food consumption in both sexes persisted at 300 mg/kg to the end of the study. Decreased weight gain was observed at 100 mg/kg in females. Despite the reduction of the high dose to 300 mg/kg, 4 animals were euthanized (including 2 toxicokinetic satellite animals), due to significant weight loss and adverse clinical signs, including piloerection, decreased activity, and hunched stance. Hematological changes were not evident at the 3-week evaluation, but were present after 6 weeks. Decreases in white blood cells (WBC) in both sexes and decreased platelets in females occurred at 500→300 mg/kg. The decrease in WBC was due mainly to decreased lymphocytes. Decreased circulating reticulocytes were observed in males at 500→300 mg/kg and in females at ≥30 mg/kg. Decreased proliferating erythrocytes were observed in the bone marrow at ≥30 mg/kg. Red blood cell (RBC) changes were not observed at 30 or 100 mg/kg, therefore effects on reticulocytes and proliferating erythroid cells at 30 mg/kg were not considered adverse. Quantitative bone marrow assessment indicated cytotoxicity in both sexes at 500→300 mg/kg in both the erythroid and myeloid lineage, with a greater effect on the erythroid lineage. Total erythroid

090177e180ac49ac\1.0\Approved\07-May-2009 14:41

numbers were also decreased in both sexes at 100 mg/kg. Absolute lymphocyte numbers were not decreased in the bone marrow of treated animals. Bone marrow macrophages were increased 14- to 23- fold at 500→300 mg/kg, however, no necrosis was observed microscopically. Changes in clinical chemistry were attributed to reduced food intake or were not considered adverse. Atrophy of the gastrointestinal tract at 500→300 mg/kg was attributed to reduced food consumption. Minimal focal erosion of the stomach was present at 100 mg/kg and 500→300 mg/kg. Atrophy of reproductive organs in males and females was observed at 500→300 mg/kg. Testicular spermatid retention was observed at 100 mg/kg and 500→300 mg/kg (accompanied by oligospermia). Foamy macrophages were present in the lung at 500→300 mg/kg. Single cell necrosis was observed in the liver at 500→300 mg/kg, accompanied by gammaglutamyl transferase (GGT) activity. Thinning of the growth plate in the joint at 500→300 mg/kg was attributed to decreased growth and effects in the Harderian gland were considered irrelevant for humans. The NOAEL was considered to be 30 mg/kg.

1.2.1.2. 6-Week Pivotal Dog Study

In a six week definitive general toxicology study, Beagle dogs were administered 30, 100, or 300 mg/kg PNU-100480 in 20% SBE-CD. No adverse effects were observed at any dose, hence the NOAEL was 300 mg/kg.

Systemic exposure ($AUC_{[0\text{-}t]}$)of PNU-100480 combined with active metabolites PNU-101603 and PNU-101244 at the NOAEL in rats and dogs was 31.9 µg·h/mL and 291 µg h/mL, respectively. Target organs identified in the rat in these repeat-dose toxicity studies were bone marrow (myeloid and erythroid depletion), reproductive organs (atrophy and spermatid retention), the lung (foamy macrophages), gastrointestinal tract (atrophy and stomach erosion), and the liver. Threshold concentrations of PNU-100480 associated with key responses in rats and dogs can be found in Table 1.

1.2.1.3. Relationship of Findings to Pharmacokinetics

Combined PNU-100480 and metabolite (PNU-101603 and PNU-101244) exposure in rats and dogs, as defined by C_{max} and $AUC_{(0\text{-tlast})}$, increased with increasing dose over the dose ranges tested. In studies with repeated daily administration, there were no sex differences or differences when comparing combined parent and metabolite exposure parameters on Day 1 to later days in the study. However, exposure of female rats to the parent compound PNU-100480 was approximately 2-fold higher than in males. Exposure to the metabolite PNU-101603 ranged from 4.5- to 16-fold of exposure to parent, while exposure to the metabolite PNU-101244 was generally less (~12% to 72%) of parent in rats, with the exception of males at 30 mg/kg (170%). Similar ratios were noted in dogs. The clinical exposure limit is set using combined exposure values of the parent compound and the 2 active metabolites at the LOAEL in the rat (100 mg/kg). Given the similar ratios of exposure to parent and metabolite in rats and dogs at various doses, the threshold plasma concentrations of PNU-100480 are expressed combined with those of active metabolites at doses associated with key responses and can be found in Table 1.

090177e180ac49ac\1.0\Approved\07-May-2009 14:41

Table 1. Combined Concentrations of PNU-100480, PNU-101603, and PNU-101244 Associated with Key Responses

Key Response(s)	Dose (mg/kg)	C_{max}[a] (µg/mL)	$AUC_{(0\text{-}tlast)}$[a] (µg•h/mL)	Exposure Margin[b]
Single-Dose Toxicity Studies				
Dog (1/sex/dose)				
NOAEL	**200**	16.5	222	1/2
Repeat-Dose Toxicity Studies				
**14-day toxicity study in rats (5/sex/dose)**				
Weight loss (F), decreased weight gain (M)	200	17.9	106	1/1
Weight loss (F), decreased weight gain (M),	500	32.6	412	2/3
Death/euthanasia (F), weight loss	1000	44.4[c]	723[c]	3/6
**6-week toxicity study in rats (10/sex/dose)**				
NOAEL	**30**	8.47	31.9	0.6/0.3
Spermatid retention, ↓ erythroid (bone marrow), gastric erosion	100	15.0	122	1/1
Moribund euthanasia, piloerection, decreased activity, hunched stance, abdomen distended, weight loss, decreased food consumption, ↓ WBC, ↓ platelets, ↓ reticulocytes, ↓ fibrinogen, ↓ PT time, ↓ erythroid and myeloid, ↑ marcophages (bone marrow), foamy macrophages (lung), spermatid retention, reproductive organ atrophy, individual cell necrosis (liver), ↑GGT, gastric erosion, villous atrophy, acinar cell depletion (pancreas), ↓ secretory granules, ↑ single cell necrosis (salivary gland)	500→300	32.2	412	2/3
**6-week toxicity study in dogs (3/sex/dose)**				
	30	9.32	32.8	0.6/0.3
	100	35.6	139	2/1
NOAEL	**300**	62.3	291	4/2

NOAEL doses are emboldened.
NOAEL = No Observed Adverse Effect Level; C_{max} = Maximum (peak) observed drug concentration; $AUC_{(0\text{-}tlast)}$ = AUC from 0 to time t last post-dose; F = Female; M = Male; WBC = White blood cell count; PT = prothrombin time, GGT = gammaglutamyl transferase
[a] In repeat-dose studies, AUC and C_{max} values indicate mean serum concentrations. Reported values were obtained near termination, or as specified.
[b] C_{max}/AUC, exposure margins are based on total plasma concentrations at the clinical exposure threshold (combined parent and metabolites; 15 µg/mL and 122 µg·h/mL).
[c] Male values, all females died or were euthanized.

1.2.1.4. Target Organ Toxicity

Based on the nonclinical studies conducted, the bone marrow, reproductive organs, lung, stomach, and liver have been identified as potential targets in the rat. The targets listed below were identified in the 6-week pivotal rat study. Target organs were not identified in the dog. Other findings related to administration of PNU-100480 included effects on the joint and Harderian gland.

090177e180ac49ac\1.0\Approved\07-May-2009 14:41

1.2.1.4.1. Hematopoietic System

Administration of PNU-100480 produced myelosuppression in rats, characterized by decreases in WBC (both sexes) and platelets at 500→300 mg/kg, decreases in reticulocytes (females) at ≥30 mg/kg, which correlated with flow cytometric data that showed decreases in the erythroid lineage at ≥100 mg/kg and in the myeloid lineage at 500→300 mg/kg. The reduction of WBCs was due primarily to the reduction in lymphocytes, however, in the bone marrow, the absolute number of lymphocytes was not affected. Absolute proliferating erythrocytes were decreased statistically significantly at 30 mg/kg in males (20%) and at 500→300 mg/kg in males and females (51% and 46%, respectively), however, the decrease in both reticulocytes (19%) and proliferating erythroid cells (20%) at 30 mg/kg was not considered adverse due to the small magnitude of the change and the lack of effect on red blood cell parameters at 30 and 100 mg/kg. Individual females at 500→300 mg/kg had nucleated red blood cells or unusual large polychromatophilic cells, while males had an increase in MCHC at 500→300 mg/kg. Changes in hematologic parameters were not present at the 3-week mid-study evaluation.

Microscopically, minimal to mild bone marrow hypocellularity was observed at 500→300 mg/kg. Increased macrophages (14- to 23-fold) were observed in the bone marrow smears at 500→300 mg/kg, however no necrosis was observed microscopically. Increases in macrophage numbers may be secondary to changes in cell turnover in the bone marrow.

At 500→300 mg/kg, fibrinogen was decreased to 66-78% of control values, while prothrombin time was increased (1.09- to 1.64-fold of controls).

The myelosuppression may in part be caused by decreased food intake as seen in food restricted rats.[20, 21] Linezolid is known to produce reversible myelosuppression in rats and dogs, generally at doses that are associated with decreased food consumption, weight loss, and decreased weight gain. In Phase 3 comparator controlled clinical trials (up to 600 mg every 12 hours for 28 days), the percentage of adult patients treated with substantially low platelet counts treated with LZD was 2.4% and 1.5% with a comparator. The decreases in platelet counts were reversible in most patients.[22] Myelosuppression may be monitored in the clinic by hematological parameters. The NOAEL for myelosuppression by PNU-100480 in rats was 30 mg/kg.

1.2.1.4.2. Reproductive Organs

Spermatid retention in the rat testis, characterized by retention of elongated spermatids in stage IX-XII tubules, was observed in 8 males at 500→300 mg/kg and 1 male at 100 mg/kg. This change was generally accompanied by minimal oligospermia in the epididymis at 500→300 mg/kg. The no observed effect level (NOEL) for spermatid retention was 30 mg/kg.

Atrophy of the epididymis, prostate, and seminal vesicle was observed in 10, 8, and 7 male rats respectively at the high dose of 500→300 mg/kg. Atrophy of the ovary, uterus, vagina and cervix was observed in all females at 500→300 mg/kg. Mammary gland atrophy was

090177e180ac49ac\1.0\Approved\07-May-2009 14:41

observed in 5 males and 10 females at 500→300 mg/kg. The NOEL for reproductive organ atrophy was 100 mg/kg.

Absolute and relative organ weights (ovary, epididymis) were lower than control values (27% to 55%) at 500→300 mg/kg. Prostate weights (absolute and relative) were 31% lower than control values at 30 mg/kg, and 8% to 14% lower than control values at 100 mg/kg. At 500→300 mg/kg, absolute and relative prostate weights were 61% and 39% lower than controls, respectively. The decrease in prostate weight at 30 and 100 mg/kg was not considered adverse, as it was not accompanied by histopathological correlates at these doses.

Atrophy of the reproductive organs after PNU-100480 administration at 500→300 mg/kg may be due to the effects of decreased feed intake, decreased weight gain, and weight loss observed at that dose. In addition, the 1 male rat at 100 mg/kg with spermatid retention had a precipitous drop in food consumption and body weight during the last week of the study. In a recent food restriction study,[23] male rats fed 66% of controls starting at 8 weeks of age had spermatocyte degeneration, slightly decreased epididymal weights, markedly reduced seminal vesicle weights, and decreased ventral prostate weights. Plasma testosterone was significantly decreased to 24% of controls.

1.2.1.4.3. Lung

In the rat lung, an increase in the severity to both minimal and mild (both sexes) and incidence (females only) of alveolar foamy macrophages was observed at 500→300 mg/kg. The foamy macrophages were observed in other groups including controls as minimal and in 2 to 3 rats per sex per dose. At the high dose, foamy macrophages were mild in 2 males and 3 females, and minimal in 2 males and 4 females. The NOAEL for foamy macrophages in the lung was 100 mg/kg.

1.2.1.4.4. Gastrointestinal System

In the rat stomach, minimal focal erosion was observed in 2 males and 4 females at 500→300 mg/kg and in 1 male at 100 mg/kg. The NOAEL for erosion was 30 mg/kg.

Superficial gastric erosions have been observed in rats given minimal to severe feed restricted diets for two weeks.[21] While mean food consumption was not decreased in males at 100 mg/kg, the 1 male with gastric erosion at this dose had a precipitous drop in food consumption and body weight during the last week of the study. While the cause of this finding was not determined, the microscopic finding may be secondary to stress associated with decreased body weight. A direct or systemic effect on the stomach by PNU-100480 cannot be excluded.

Villus atrophy (2-8 females) of the small intestine (jejunum or ileum), depletion of zymogen granules in the acinar cells of the pancreas (7 males and 7 females), decreased secretory granules (4 males and 7 females) and increased single cell necrosis (6 males and 1 female) in the salivary gland were observed at 500→300 mg/kg; these findings were attributed to prolonged decreased food intake.

090177e180ac49ac\1.0\Approved\07-May-2009 14:41

Cecal enlargement without histological abnormalities was observed in rats at ≥30 mg/kg. This finding is considered to be a secondary to a bactericidal effect on cecal microflora as previously reported in rats administered antibiotics.[24]

1.2.1.4.5. Liver

In the rat liver, deposition of golden-brown pigment in Kupffer cells was observed in 6 females and single cell necrosis of hepatocytes was found in 4 females at 500→300 mg/kg. The golden-brown pigment was positive for iron stain on representative animals and interpreted as hemosiderin pigment and not considered adverse. Multifocally, slight basophilia of periportal hepatocytes, which was occasionally accompanied by mitosis of hepatocytes, was seen in 5 females at both 30 mg/kg/day and 100 mg/kg/day. The relationship of periportal basophilia of hepatocytes with treatment was uncertain because of a lack of this finding in the higher dose group. Mean GGT values were slightly but statistically significantly elevated in males and females at 500→300 mg/kg.

Liver transaminases (ALT and AST) in females were increased by approximately 2-fold, 2- to 3-fold, and 4-fold at 30, 100, and 500→300 mg/kg, respectively. This increase was not considered adverse as there were no histological correlates and LFTs can be readily monitored in the clinic. The NOAEL for the liver was 100 mg/kg.

1.2.1.4.6. Other Tissues

In the rat joint, minimal thinning of growth plates of long bones (tibia, femur) was observed in 8 males and 10 females at 500→300 mg/kg/day. This finding was considered related to decreased food intake and correlated with decreased alkaline phosphatase (ALP) values.

In the acinar epithelium of the Harderian gland, an incidence of single cell necrosis above that found in controls was observed in 3 males and 6 females at 500→300 mg/kg/day. The relevance for humans is not clear as humans do not have a Harderian gland.

Reductions in absolute organ weights and increases in relative organ weights (liver, kidney, adrenal, heart, brain, spleen, and thymus) at 100 and/or 500→300 mg/kg were considered to be secondary to changes in weight gain at those doses.

Lymphoid depletion was observed in the thymus (7 males and 10 females), spleen (8 females), mesenteric lymph node (6 females), inguinofemoral lymph node (2 males and 6 females) and gut-associated lymphoid tissue (GALT, 1 female) at 500→300 mg/kg/day. GALT of 7 females from 500→300 mg/kg/day group was not present in the intestinal tissue sections and this was attributed to lymphoid depletion. Lymphoid depletion of these tissues was most likely a stress-related change. Absolute and relative thymus weights were decreased by approximately 20% in females at 100 mg/kg and by 52% to 85% in males and females at 500→300 mg/kg. Absolute and relative spleen weights were decreased by 37% to 62% at 500→300 mg/kg.

090177e180ac49ac\1.0\Approved\07-May-2009 14:41

1.2.1.5. Genotoxicity

PNU-100480 was assessed in a series of genetic toxicology assays consisting of the microbial reverse mutation, in vitro cytogenetic (human lymphocyte), and in vivo rat micronucleus assays. All in vitro tests were conducted with and without exogenous metabolic activation using concentrations up to those limited by cytotoxicity or insolubility. PNU-100480 was not genotoxic in either in vitro or in vivo assays (Table 2).

Table 2. Genotoxicity Studies Conducted with PNU-100480

Type of test	Lot Number	Test System	Metabolic Activation[a]	Dose/ Concentration	Results
Microbial reverse mutation assay	GR01567	*Salmonella typhimurium* and *Escherichia coli*	Without and With	0.00050-5.0 mg/plate	(–)
Cytogenetics assay	GR01567	Human lymphocytes (in vitro)<br>3-hour<br>24 hour<br>3 hour	 Without<br>Without<br>With	34-544 µg/mL<br>68-544 µg/mL<br>136-1088 µg/mL	 (–)<br>(–)<br>(–)
Cytogenetics assay	082075	Rat bone marrow cells (in vivo)[b]	N/A	500, 1000, 2000 mg/kg	(–)

N/A – Not applicable.
a Aroclor-induced rat liver S-9 fraction from male SD rats.
b The mean C_{max} and $AUC_{(0\text{-}tlast)}$ values (males and females) for PNU-100480 on Day 2 in rats given 2000 mg/kg were 74/98.3 µg/mL and 981/1289 µg·h/mL, respectively.

Carcinogenicity, reproductive, and developmental toxicity studies with PNU-100480 have not been conducted.

1.2.2. Pharmacokinetic Summary

Data from the in vitro and in vivo ADME studies indicate that PNU-100480 is rapidly absorbed in rats and dogs. Following intravenous (IV) administration of PNU-100480, plasma clearance of PNU-100480 in rats and dogs was high. The steady-state volume of distribution was greater than total body water in the corresponding species. Based on the exposure profiles of the combined parent and metabolites, pharmacokinetics of PNU-100480 in rat and dog are characterized by moderate clearance (CL), moderate volume of distribution (Vss), moderate half life (t1/2), and high oral bioavailability (%F). The systemic exposures of each analyte as well as the combined parent and metabolites increased with increasing dose over the dose range evaluated in nonclinical toxicology studies in these species.

Absorption of PNU-100480 following single dose oral administration was high, with mean absolute bioavailability >100% in rats and 50% to 74% in dogs based on the combined exposure profiles.

Plasma protein binding of PNU-100480, PNU-101603, and PNU-101244 was low in mouse, rat, dog, and human with the unbound fraction ranging from 48% to 76%, 79% to 102%, and 74% to 97%, respectively. (Table 3) Species differences in binding were not observed, and

090177e180ac49ac\1.0\Approved\07-May-2009 14:41

binding was concentration-independent across the species evaluated. As a result, all exposure levels stated are total values.

Table 3. Summary of Plasma Protein Binding (% Free) and RBC Distribution (Kp) for PNU-100480, PNU-101603, and PNU-101244

Compound	Species	Concentration (μg/mL)	Human	Mouse	Rat	Dog
PNU-100480	Protein Binding (% free)	1	52	60	48	76
		5	55	58	48	66
	RBC Distribution	1	1.1	ND	ND	ND
PNU-101603	Protein Binding (% free)	1	96	100	94	86
		5	82	85	79	86
	RBC Distribution	1	0.89	ND	ND	ND
PNU-101244	Protein Binding (% free)	1	94	95	88	97
		5	78	80	74	83
	RBC Distribution	1	0.97	ND	ND	ND

RBC= Red blood cell; ND= Not determined

In vivo, PNU-100480 undergoes extensive metabolism to form the major sulfoxide metabolite (PNU-101603) and the secondary sulfone metabolite (PNU-101244). These metabolites exhibited similar pharmacological activity compared to the parent compound. Qualitatively similar metabolite profiles were observed in vitro in rat, dog, and human liver microsomes and hepatocytes. All PNU-100480 metabolites observed in human in vitro systems were also present in 1or more of the in vitro evaluated nonclinical species. PNU-100480 is metabolically cleared by CYP3A4, flavin-containing monooxygenase (FMO) 1 and FMO3. Following a 10 mg/kg IV dose of PNU-100480, metabolite PNU-101603 accounted for 90% and 48% of the dose that was excreted in urine in rats and dogs, respectively. These data suggest that renal and biliary excretions of unchanged drug are not significant clearance pathways and that metabolism is expected to be the major clearance mechanism for PNU-100480.

The relative contributions to the total combined AUC of PNU-100480, PNU-101603, and PNU-101244 are 16.9%, 78.9%, and 6.0%, respectively, following a 10 mg/kg IV dose of PNU-100480 to rats; and 22.1%, 73.9%, and 3.4%, respectively, following a 10 mg/kg IV dose of PNU-100480 to dogs. In vitro, PNU-100480 is metabolically cleared by CYP3A4, FMO1, and FMO3.

Following a 10 mg/kg IV dose of PNU-100480 to rats and dogs, 1.1% and 11% of the dose was excreted in urine as unchanged drug. The metabolite PNU-101603 accounted for

090177e180ac49ac\1.0\Approved\07-May-2009 14:41

90% and 48% of the dose in rats and dogs, respectively, whereas PNU-101244 accounted for 4.9% and 2.3% of the dose.

In humans, PNU-100480 is predicted to have high CL (53 mL/min/kg), a high Vss (25.3 L/kg), a moderate t1/2 (5.5 h), and bioavailability of 100%. The nonclinical PK/PD relationship of PNU-100480 was evaluated in an established murine lung infection model using *M. tuberculosis* H37Rv following oral administration of PNU-100480 at 25-100 mg/kg (5 days/week, 4 to 8 weeks). Based on the PK/PD and the predicted human pharmacokinetic parameter values, it is projected that a dosing regimen of 410 mg to 490 mg once daily is anticipated to achieve the targeted therapeutic AUC of 1.8 to 2.2 µg·h /mL for PNU-100480 alone, based on Day 1 and Day 24 exposures in mice, respectively. Based on Day 1 and Day 24 exposure values in mice, this corresponds to combined exposures of 12 to 29 µg·h /mL, respectively, for the sum of PNU-100480, PNU-101603, and PNU-101244. These targeted exposure values were estimated based on exposures from the 100 mg/kg dosing group on Day 24 assuming linear kinetics.

1.2.2.1. Pharmacokinetics/Pharmacodynamics

The pharmacokinetic/pharmacodynamic (PK/PD) relationship of PNU-100480 was evaluated in a murine lung infection model using *M. tuberculosis* H37Rv. Treatment with PNU-100480, as a single agent, prevented splenomegaly and resulted in a significant reduction in mean colony forming unit (CFU) count from baseline at the lowest dose of 25 mg/kg, and exposures at 25 mg/kg were extrapolated from those determined at 100 mg/kg. It is presumed that area under the curve/minimum inhibitory concentration (AUC/MIC) is a major determinant to produce the intended pharmacologic effect based on the dosing paradigm (5 days/week). Based on the PK/PD and predicted human PK values, a dosing regimen of 410-490 mg once daily is anticipated to achieve the targeted therapeutic exposure of PNU-100480.

Table 4. Projected Steady-State Cmax, Cmin and AUC of PNU-100480, PNU-101603, and PNU-101244 in Human after 410 mg Assuming Accumulation of PNU-101603 and PNU-101244 as Observed on Days 1 and 24 in Mouse Efficacy Study

	**Day 1**			**Day 24**		
	Cmax (µg/mL)	Cmin (µg/mL)	AUC (µg•h/mL)	Cmax (µg/mL)	Cmin (µg/mL)	AUC (µg•h/mL)
PNU-100480	0.17	0.014	1.8	0.21	0.017	2.2
PNU-101603	0.87	0.070	9.2	2.3	0.19	25
PNU-101244	0.061	0.0049	0.64	0.23	0.018	2.4
Sum	1.1	0.089	12	2.8	0.23	29

Cmax = Maximum (peak) observed drug concentration; AUC = Area under concentration-time curve;
Sum = Exposure to PNU-100480 and metabolites (PNU-101603 and PNU-101244) combined.

090177e180ac49ac\1.0\Approved\07-May-2009 14:41

1.2.2.2. Toxicokinetics

In toxicology studies with rats and dogs, gender-related disposition was observed only in rats for PNU-100480, and PNU-101244; higher systemic exposures were observed in female rats for PNU-100480, while exposure to PNU-101244 was higher in males. Exposure to the predominant metabolite PNU-101603 was similar in both sexes, hence combined exposure to parent and metabolites was also similar in both sexes. Systemic exposure of PNU-100480, PNU-101603, and PNU-101244, as well as the sum exposure of all three analytes, increased in a dose-related manner up to 300 mg/kg/day with no time-dependent change in exposure observed over the treatment duration in both rats and dogs.

2. STUDY OBJECTIVES AND ENDPOINTS

2.1. Objectives

- To evaluate the safety and tolerability of escalating single oral doses of PNU-100480 administered as extemporaneously prepared (EP) suspension in healthy adult volunteers.

- To characterize the pharmacokinetics of single oral doses of PNU-100480 in healthy adult volunteers with regard to the parent drug and its metabolites.

- To characterize the effect of food on the pharmacokinetics of a single oral dose of PNU-100480.

- To characterize the bactericidal activity in blood of orally administered PNU-100480 against intracellular *M. tuberculosis* in relation to blood concentrations of PNU-100480 and its metabolites.

2.2. Endpoints

- Standard FIH safety assessments including assessment of AEs, laboratory tests, ECGs, and vital signs.

- Plasma concentrations of PNU-100480 and its metabolites will be used to determine PK parameters (AUC_{inf}, AUC_{last}, C_{max}, Tmax and terminal half-life) as data permit. Apparent oral clearance (CL/F) and apparent volume of distribution (Vz/F) will also be determined for PNU-100480.

- Bactericidal activity in whole blood (measured as log reduction in CFU) against intracellular *M. tuberculosis* relative to drug and metabolite PK.

090177e180ac49ac\1.0\Approved\07-May-2009 14:41

3. STUDY DESIGN

3.1. Study Overview

This study is a randomized, investigator-blind, subject-blind, sponsor-open, placebo substitution, sequential cohort, single dose escalation study of PNU-100480. In Cohort 1, each subject will receive 2 escalating single oral doses of PNU-100480 or placebo (Periods 3 periods, fasted). In Cohort 2, each subject will receive 2 escalating single oral doses of PNU-100480 or placebo (3 periods, fasted) and an open label dose of PNU-100480 under fed conditions (Period 4).

Subjects will undergo screening procedures within 28 days prior to dosing. Eligible subjects who meet the entry criteria will be admitted to the Clinical Research Unit (CRU) on Day 0 of each period and confined for at least 48 hours after dosing with either PNU-100480 or placebo.

Approximately 18 healthy volunteers, 18 to 55 years of age, males and females (women of non-childbearing potential only) will be enrolled in 2 cohorts with 9 subjects for each cohort and assigned to randomized treatment sequences within each cohort.

In each period, each subject will receive a single oral dose of either placebo or PNU-100480 under fasted conditions. Subjects in Cohort 2 will also receive an open label, single oral dose of PNU-100480 under fed conditions. For this period only, subjects will complete a high fat meal prior to dosing.

Planned doses may be adjusted up or down (within the planned limits) based on emerging safety and pharmacokinetic results from the previous dose period. Dose escalation will only proceed if safety of the previous cohort is deemed satisfactory, and none of the stopping criteria have been met.

Cohort 1:

Sequences	Number of Subjects	Period 1	Period 2	Period 3
1	3	Placebo	100 mg	300 mg
2	3	35 mg	Placebo	300 mg
3	3	35 mg	100 mg	Placebo

Cohort 2:

Sequences	Number of Subjects	Period 1	Period 2	Period 3	Period 4
1	3	Placebo	1000 mg	1500	600
2	3	600 mg	Placebo	1500	600
3	3	600 mg	1000 mg	Placebo	600

There will be an interval of at least 7 days between periods to allow for assessment of the preliminary safety, tolerability and PK data from the previous period.

090177e180ac49ac\1.0\Approved\07-May-2009 14:41

Whole blood samples will be collected for the planned measurement of bactericidal activity in Cohort 1, Period 3, and Cohort 2, Periods 1, 2, and 3.

Excluding the screening period for up to 28 days, the study duration for each subject will be up to approximately 35 day for Cohort 1 and up to approximately 50 days for Cohort 2.

3.2. Human Pharmacokinetic Projections

Rat was selected for the prediction of human PK parameters for two reasons: 1) the in vitro metabolic rates (disappearance of the PNU-100480) using human liver microsomes were found to be qualitatively similar to rat liver microsomes 2) as per recommendation in the white paper for "Human PK Predictions" rat is the recommended species for human PK prediction when there is no knowledge which species is more predictive for human. Human clearance, volume of distribution at steady state, and elimination half-life for PNU-100480 from rats were projected as 53 mL/min/kg, 25.3 L/kg and 5.5 hour, respectively. Human oral bioavailability is predicted to be approximately 100%, this is projected from the bioavailability observed in rat which is >100%. Based on these predicted human pharmacokinetic parameters and the PK/PD relationship, a dosing regimen of 410 mg to 490 mg QD is predicted to be efficacious.

3.3. Dose Rationale and Stopping Rules

The planned potential PNU-100480 dose range to be evaluated in this study is 35 mg to 1500 mg. These doses were selected for: 1) characterization of PK and the safety of PNU-100480 over a wide range of doses; and 2) allow for exploration of doses that would achieve the projected combined efficacious exposure of 12 - 29 µg·hr/mL (Table 4). The projected exposure of PNU-100480 (parent) at doses 35 – 1500 mg are shown in Table 5 below. The expected pharmacokinetic exposure of PNU-100480 (parent) and two active metabolites (PNU-101603 and 101244) at planned doses of 35 mg to 1500 mg and safety margins are shown in Table 6 below.

The in vitro metabolic rates of formation of two metabolites and rate of disappearance of parent drug in human appears to be qualitatively similar to mouse and rat. Therefore, the relative ratios of two metabolites and parent drug were assumed to be same in mouse as well as in human. The relative ratios of two metabolites, PNU-101603 and PNU-101244 compared to PNU-100480 observed on day 24 in mouse (murine lung infection model) are 11 and 1.1, respectively. The two metabolites (PNU-101603 and PNU-101244) and PNU-100480 have similar in vitro antibacterial potency and therefore, a combined exposure of the three analytes will be used.

The exposures are calculated assuming linear pharmacokinetics of PNU-100480 as was observed in rat and dog over a wide range of doses and adding the exposure of two active metabolites using the relative ratios as defined above. The starting dose of 35 mg is approximately $1/10^{th}$ of the HED of 338.7 mg calculated using 30 mg/kg NOAEL dose in the rat. The exposure at the starting single dose of 35 mg is also approximately $1/14^{th}$ of the exposure at projected daily dose of 410 mg to 490 mg at steady state, which is expected to yield a combined AUC and C_{max} of 29 µg·hr/mL and 2.8 µg/mL, respectively (Table 4). The

090177e180ac49ac\1.0\Approved\07-May-2009 14:41

projected efficacious AUC of 12 - 29 μg·hr/mL is projected from the murine *M. tuberculosis* lung infection model and is associated with a significant effect.

Table 5. Projected Exposures of PNU-100480 (Parent Only) after Administration of Single Dose to Humans

Dose (mg)	Cmax (μg/mL)a	AUC total (μg·h/mL)b
35	0.014	0.157
100	0.04	0.449
300	0.12	1.35
600	0.24	2.69
1000	0.40	4.49
1500	0.60	6.74

[a]C_{max} was calculated using the expression $C_{max} = (F*Dose/V)*e^{-K*Tmax}$, T_{max} = Ln (Ka/K)/(Ka-K) = 2.75 Hr
[b]Calculated using the relationship AUC=Dose*F/CL.
CL = 222,600 mL/hr for a 70 kg human, F =1, V= 1771 L for a 70 kg human, Ka was assumed as 0.8 hr^{-1}

Table 6. Projected Exposures and Safety Margin of Parent (PNU-100480) and Two Metabolites (PNU-101603 and PNU-101244) Relative to Rat 100 mg/kg Dose Exposures after Administration of Single Dose of Humans

Dose (mg)	Combined Cmax (μg/mL)[a]	Safety Margin Using Total Cmax[b]	Combined AUC Total (μg·h/mL)[c]	Safety Margin Using Total AUC[d]
35	0.18	81.94	2.06	59.23
100	0.52	28.68	5.88	20.73
300	1.57	9.56	17.65	6.91
600	3.14	4.78	35.31	3.45
1000	5.24	2.87	58.85	2.07
1500	7.86	1.91	88.27	1.38

[a] Combined Cmax was calculated using relative ratio of PNU-101603 and PNU-101244 to PNU-101480 (parent) as 11 and 1.1, respectively.
[b] Escalation limit for Cmax is 15 μg/mL based on the mean combined concentrations of two metabolites and parent at 100 mg/kg dose in rat (combined sexes).
[c] Combined AUC was calculated using relative ratio of PNU-101603 and PNU-101244 to PNU-101480 (parent) as 11 and 1.1, respectively.
[d] Escalation limit for AUC is 122 μg·h/mL based on the mean combined AUC of two metabolites and parent at 100 mg/kg dose in rat (combined sexes).

Since all 3 analytes are active and have similar pharmacologic activity, the proposed PK stopping criteria are based on the combined exposure of 3 analytes. The mean combined AUC and Cmax of the 3 analytes observed in rats (combined sexes) at 100 mg/kg are

090177e180ac49ac\1.0\Approved\07-May-2009 14:41

122 μg·h/mL and 15 μg/mL, and will be used as the stopping criteria. If either of the exposure limits is reached, we will not proceed above that dose. Depending upon the total observed exposure, doses may be adjusted up or down to meet the exposure, but the maximum dose would not go above the exposure limit set for this study (AUC and Cmax of 122 μg·h/mL and 15 μg/ml, respectively).

The predicted exposure in humans at maximum dose of 1500 mg is approximately 1.5 fold less than rat exposure at the 100 mg/kg dose. Exposure at 100 mg/kg was used to set the stopping criteria as well as the safety margins because the adverse events observed at this dose were due to long term exposure to the drug, are monitorable in the clinic, and are not serious in nature. Stopping criteria based on combined exposure of all 3 analytes will be utilized to guide dose escalation. The main potential toxicity of PNU-101480 and its metabolites is related to inhibition of mitochondrial protein synthesis (MPS), an effect similar to Linezolid. The toxicity is delayed, readily monitored in clinic and reverses on drug discontinuation. As the MPS IC50 values of PNU-100480 and its metabolites are similar, it suggests that the toxicities of the parent and its metabolites due to MPS inhibition would be similar and additive. Therefore, combined exposure of PNU-100480 and its metabolites are proposed as stopping criteria.

Dose escalation will be contingent on the absence of all of the following:

1. Any serious adverse event in a subject receiving active drug.
2. Severe adverse events in the same organ system in ≥2 subjects receiving active drug in the same period.
3. Clinically significant ECG or BP/HR abnormalities in ≥2 subjects receiving active drug in the same period.
4. Clinically significant laboratory abnormalities in ≥2 subjects receiving active drug in the same period.
5. Other findings that, at the discretion of the investigator, indicate dose escalation should be halted.

The decision to proceed to the next higher dose will be made jointly by the sponsor and the investigator after careful review of available safety and tolerability, and pharmacokinetic information from previous cohorts and dosing periods. There will be an interval of at least 7 days between periods in Cohort 1 and 2 to allow assessment of the preliminary available safety and tolerability data.

The collection times for vital sign measurements, electrocardiograms (ECGs), safety labs, pharmacokinetic samples and the washout period may also be modified based on emerging safety and/or pharmacokinetic data. Duration of telemetry monitoring may be increased as appropriate if a clinically significant (as determined by the investigator) change in heart rate or other abnormal cardiovascular findings occurs within the first 12 hours after dosing.

090177e180ac49ac\1.0\Approved\07-May-2009 14:41

Physical examinations, ECGs, vital sign measurements, and clinical laboratory tests will be performed and adverse events will be monitored throughout the study to assess safety and tolerability.

4. SUBJECT SELECTION

Approximately 18 healthy normal subjects, 18 to 55 years of age, males and females (women of non-childbearing potential only) will be enrolled into 2 cohorts, with 9 subjects for each cohort.

This study can fulfill its objectives only if appropriate subjects are enrolled. The following eligibility criteria are designed to select subjects for whom protocol treatment is considered appropriate. All relevant medical and non-medical conditions should be taken into consideration when deciding whether this protocol is suitable for a particular subject.

4.1. Inclusion Criteria

Subjects must meet all of the following inclusion criteria to be eligible for enrollment into the study:

1. Healthy male and/or female (women of non-childbearing potential only) subjects between the ages of 18 and 55 years, inclusive (Healthy is defined as no clinically relevant abnormalities identified by a detailed medical history, full physical examination, including blood pressure and pulse rate measurement, 12-lead ECG and clinical laboratory tests).

2. Body Mass Index (BMI) of 18 to 30 kg/m^2; and a total body weight >50 kg (110 lbs). A BMI lower limit of 17.5 kg/m^2 may be rounded up to 18.0 kg/m^2; a BMI upper limit of 30.5 kg/m^2 may be rounded down to 30.0 kg/m^2 and will be acceptable for inclusion.

3. An informed consent document signed and dated by the subject or a legally acceptable representative.

4. Subjects who are willing and able to comply with scheduled visits, treatment plan, laboratory tests, and other study procedures.

4.2. Exclusion Criteria

Subjects presenting with any of the following will not be included in the study:

1. Evidence or history of clinically significant hematological, renal, endocrine, pulmonary, gastrointestinal, cardiovascular (including hypertension), hepatic, psychiatric (including history of depression or bipolar disorder), neurologic, or allergic (including drug allergies, but excluding untreated, asymptomatic, seasonal allergies at time of dosing) disease or clinical findings at screening.

090177e180ac49ac\1.0\Approved\07-May-2009 14:41

2. Any condition possibly affecting drug absorption (eg, gastrectomy).

3. A positive urine drug screen.

4. History of regular alcohol consumption exceeding 7 drinks/week for females or 14 drinks/week for men (1 drink = 5 ounces (150 mL) of wine or 12 ounces (360 mL) of beer or 1.5 ounces (45 mL) of hard liquor) within 6 months of screening.

5. History or evidence of habitual use of tobacco- or nicotine-containing products within 3 months of screening or a positive urine or blood cotinine screen.

6. Treatment with an investigational drug within 30 days or 5 half-lives preceding the first dose of study medication.

7. Participation in a previous cohort in this study.

8. Antibiotic treatment within 14 days prior to dosing.

9. Hospitalization for any reason within 90 days prior to dosing, at the discretion of the investigator.

10. 12-lead ECG demonstrating QTc >450 msec at screening. If QTc exceeds 450 msec, the ECG should be repeated two more times and the average of the three QTc values should be used to determine the subject's eligibility.

11. Use of prescription or nonprescription drugs and dietary supplements within 7 days or 5 half-lives (whichever is longer) prior to the first dose of study medication. Herbal supplements and hormone replacement therapy must be discontinued 28 days prior to the first dose of study medication. As an exception, acetaminophen/paracetamol may be used at doses of up to 1 g/day. Nasal decongestants are excluded, except for those solutions containing only saline. Limited use of other non-prescription medications that are not believed to affect subject safety or the overall results of the study may be permitted on a case-by-case basis following approval by the sponsor.

12. Blood donation of approximately 1 pint (500 mL) within 56 days prior to dosing.

13. Consumption of grapefruit or grapefruit-related citrus products within 7 days prior to the first dose of study medication.

14. History of hypersensitivity to or intolerance of linezolid.

15. Diarrhea lasting 7 days or more in the period immediately prior to dosing.

16. Other severe acute or chronic medical or psychiatric condition or laboratory abnormality that may increase the risk associated with study participation or investigational product administration or may interfere with the interpretation of study results and, in the judgment of the investigator, would make the subject inappropriate for entry into this study.

17. Pregnant or nursing females; females of childbearing potential.

090177e180ac49ac\1.0\Approved\07-May-2009 14:41

18. Unwilling or unable to comply with the Lifestyle guidelines described in this protocol.

4.3. Randomization Criteria

Subjects will be assigned a unique PIMS number at time of screening. This identifying number will be retained throughout the duration of study participation. Subjects will be randomized into the study provided they have satisfied all subject selection criteria. A computer generated randomization schedule will be used to assign subjects to the treatment sequences.

4.4. Life Style Guidelines

4.4.1. Meals and Dietary Restrictions

- Subjects must abstain from all food and drink (except water) at least 4 hours prior to any safety laboratory evaluations and 8 hours prior to the start of pharmacokinetic sample collections (except for Cohort 2, Period 4 described below). Water is permitted until 1 hour prior to study medication administration.

- Water may be consumed without restriction beginning 1 hour after dosing. Non-caffeinated drinks (except grapefruit or grapefruit-related citrus fruit juices – see below) may be consumed with meals and the evening snack.

- Lunch will be provided approximately 4 hours after dosing.

- Dinner will be provided approximately 9 to 10 hours after dosing.

- An evening snack will be permitted up to 2200 hours.

- Subjects will not be allowed to eat or drink grapefruit or grapefruit-related citrus fruits (eg, Seville oranges, pomelos) from 7 days prior to the first dose of study medication until collection of the final pharmacokinetic blood sample of each period.

- Subjects will not be allowed to eat cheese from Day 0 of each period until collection of the final pharmacokinetic blood sample of each period.

- While confined, the total daily nutritional composition should be approximately 50% carbohydrate, 35% fat and 15% protein. The daily caloric intake per subject should not exceed approximately 3200 kcal.

- FED Conditions for Cohort 2, Period 4: Following an overnight fast of at least 10 hours, subjects should start breakfast 25 minutes prior to administration of the drug product. The breakfast will be consumed over a 20-minute period with PNU-100480 administered within 5 minutes after completion of the meal. The drug product should be administered with 240 mL (8 fluid ounces) of water. No food will be allowed for at least 4 hours postdose. Water can be allowed as desired except for

090177e180ac49ac\1.0\Approved\07-May-2009 14:41

one hour before and after drug administration. The breakfast will be a high-calorie/high-fat test meal consisting of two eggs fried in butter, two strips of bacon, two slices of toast with butter, four ounces of hash brown potatoes and eight ounces of whole milk.

4.4.2. Alcohol, Caffeine and Tobacco

- Subjects will abstain from alcohol for 24 hours prior to the start of dosing until collection of the final pharmacokinetic sample of each study period.
- Subjects will abstain from caffeine-containing products for 24 hours prior to the start of dosing until collection of the final pharmacokinetic sample of each study period.
- Subjects will abstain from the use of tobacco- or nicotine-containing products prior to dosing through the end of the study.

4.4.3. Activity

- Subjects will be confined to the procedure room for the first 4 hours after dosing on Day 1 during continuous ECG monitoring, except to use the bathroom. After this, if the equipment setup allows, subjects may be ambulatory during the telemetry period, but should not engage in strenuous activities. If equipment does not allow ambulation, appropriate accommodations will be made by the study site to facilitate continuous monitoring (ie, bedside urinals should be provided to accommodate subjects' excretory needs).
- Subjects will abstain from strenuous exercise (eg, heavy lifting, weight training, calisthenics, aerobics) for at least 48 hours prior to each blood collection for clinical laboratory tests. Walking at a normal pace will be permitted.

4.4.4. Contraception

4.4.4.1. Females – Non-childbearing Potential

Female subjects of non-childbearing potential must meet at least one of the following criteria:

1. Post-menopausal females, defined as:
    - Females who are 45 to 55 years of age must be amenorrheic for at least 2 years PLUS have a serum FSH level within the laboratory's reference range for post-menopausal females.
2. Females who have a documented hysterectomy and/or bilateral oophorectomy.

090177e180ac49ac\1.0\Approved\07-May-2009 14:41

All other female subjects (including females with tubal ligations and females that do NOT have a documented hysterectomy) will be considered to be of childbearing potential and are excluded from participation.

4.4.4.2. Males

It is required that all male subjects use one of the following methods of contraception from the first dose of study medication and until 28 days after dosing:

1. Abstinence.

2. Use of condom for males with a vasectomy for more than 6 months.

3. Male subjects who have not had a vasectomy for more than 6 months must use a condom. In addition, such a male subject should be instructed that, unless his female partner has had a tubal ligation, hysterectomy, or bilateral oophorectomy or is post-menopausal, his female partner should use another form of contraception from the time of the first dose of study medication until 28 days after dosing. Such other forms of contraception include an IUD, spermicidal foam/gel/film/cream/suppository, diaphragm with spermicide, oral contraceptive, injectable progesterone, or subdermal implant.

090177e180ac49ac\1.0\Approved\07-May-2009 14:41

5. STUDY TREATMENTS

5.1. Allocation to Treatment

Subjects will be given a unique PIMS ID that will be recorded in PIMS. Then, prior to dosing, a randomization number will be allocated. This number will be retained throughout the study and will correspond to a treatment schedule determined by a Pfizer generated randomization code. It will also appear on the study medication containers.

This trial will be conducted in 2 cohorts, with 3 cross-over periods in Cohort 1, and 4 cross-over periods in Cohort 2. There will be 9 subjects in each cohort. Subjects in each cohort will be randomly assigned to 1 of 3 treatment sequences as follows, each receiving 2 doses of PNU-100480 and 1 dose of placebo. For Cohort 2, Period 4 all subjects will receive a single oral dose of PNU-100480 following a high calorie, high fat meal.

Subjects who discontinue for non-safety reasons may be replaced at the discretion of the clinical investigator in consultation with the sponsor. Subjects who drop out for safety or tolerability reasons will not be replaced. Replacement subjects will be allocated to the same treatment sequence as the subject who discontinued.

Table 7. Treatment Allocation

Cohort	Sequences	Number of Subjects	Period 1	Period 2	Period 3	Period 4 (Fed)
1	1	3	Placebo	100 mg	300 mg	-
1	2	3	35 mg	Placebo	300 mg	-
1	3	3	35 mg	100 mg	Placebo	-
2	1	3	Placebo	1000 mg	1500	600 mg
2	2	3	600 mg	Placebo	1500	600 mg
2	3	3	600 mg	1000 mg	Placebo	600 mg

5.2. Breaking the Blind

This will be a subject- and investigator-blinded, sponsor-open study.

At the initiation of the study, the study site will be instructed on the method for breaking the blind. The method will be either a manual or electronic process. Blinding codes should only be broken in emergency situations for reasons of subject safety. When the blinding code is broken, the reason must be fully documented.

Blood specimens will be obtained from all subjects for pharmacokinetic analysis to maintain the study blind at the investigator site. Specific Pfizer personnel (eg, analytical staff, medical monitor, clinician, clinical pharmacology lead) will be unblinded to subject treatments in order to permit real-time interpretation of the safety and pharmacokinetic data; and provide information necessary to potentially alter the dose escalation sequence. Specimens from subjects randomized to placebo will not be routinely analyzed. To minimize the potential for bias, treatment randomization information will be kept confidential by Pfizer personnel and will not be released to the investigator or investigator site personnel until the conclusion of the study.

5.3. Drug Supplies

5.3.1. Formulation and Packaging

The study medication is being supplied as bulk Active Pharmaceutical Ingredient (API) and, as necessary, bulk excipients/components for extemporaneous preparation in the site Pharmacy. The study medication will be administered as an extemporaneously prepared suspension (EP-Suspension) for all doses within the range of 35 mg to 1500 mg. Placebo doses corresponding to all doses of study medication will be a suspension.

All materials will be supplied by the sponsor in bulk form for extemporaneous preparation at the site. An Extemporaneous Dispensing Record (EDR) will be provided separately to the site and will detail the preparation and dosing procedures for all doses.

090177e180ac49ac\1.0\Approved\07-May-2009 14:41

5.3.2. Preparation and Dispensing

Each dose of trial medication will be prepared by a pharmacist at the site who is unblinded to the treatment assigned and who is not responsible for the reporting or documenting of adverse events. Only qualified personnel who are familiar with procedures that minimize undue exposure to them and to the environment should undertake the preparation, handling, and safe disposal of chemotherapeutic agents.

5.3.3. Administration

Following an 8-hour fast, subjects will receive study medication at approximately 0800 hours (plus or minus 2 hours). Investigator site personnel will administer study medication with ambient temperature water to a total volume of 240 mL. In order to standardize the conditions on pharmacokinetic sampling days, all subjects will be required to refrain from lying down (except when required for blood pressure, pulse rate, and ECG measurements), eating and drinking beverages other than water during the first 4 hours after dosing.

For Cohort 2, Period 4 (fed): Following an overnight fast of at least 10 hours, subjects should start breakfast 25 minutes prior to administration of the drug product. The breakfast will be consumed over a 20-minute period with PNU-100480 administered within 5 minutes after completion of the meal. The drug product should be administered with 240 mL (8 fluid ounces) of water. No food will be allowed for at least 4 hours post dose. Water can be allowed as desired except for one hour before and after drug administration. The breakfast will be a high-calorie/high-fat test meal consisting of two eggs fried in butter, two strips of bacon, 2 two slices of toast with butter, 4 ounces of hash brown potatoes and 8 ounces of whole milk.

5.3.4. Compliance

Study treatment will be administered under the supervision of investigator site personnel.

5.4. Drug Storage and Drug Accountability

The investigator, or an approved representative, eg, pharmacist, will ensure that all investigational product is stored in a secured area, under recommended storage conditions and in accordance with applicable regulatory requirements. To ensure adequate records, all study drugs will be accounted for in the Phase 1 management system (PIMS) or via the drug accountability forms as instructed by Pfizer.

At the end of the study, Pfizer will provide instructions as to disposition of any unused investigational product. If Pfizer authorizes destruction at the study site, the investigator must ensure that the materials are destroyed in compliance with applicable environmental regulations, institutional policy, and any special instructions provided by Pfizer. Destruction must be adequately documented.

090177e180ac49ac\1.0\Approved\07-May-2009 14:41

5.5. Concomitant Medication(s)

Subjects will abstain from using prescription or nonprescription drugs, vitamins, and dietary supplements within 7 days or 5 half-lives (whichever is longer) prior to the first dose of study medication and throughout the conduct of the study. Herbal supplements and hormone replacement therapy must be discontinued at least 28 days before the first dose of study medication. Acetaminophen may be used intermittently throughout the study, but should not exceed 1 g/day on two consecutive days, unless greater quantities are deemed necessary by the investigator.

All concomitant medications taken during the study must be recorded with indication, daily dose, and start and stop dates of administration. All subjects will be questioned about concomitant medication at each clinic visit.

Medications taken within 28 days before the first dose of study medication will be documented as a prior medication. Medications taken after the first dose of study medication will be documented as concomitant medications.

6. STUDY PROCEDURES

6.1. Screening

Subjects will be screened within 28 days prior to administration of the study medication to confirm that they meet the subject selection criteria for the study. The investigator (or an appropriate delegate at the investigator site) will obtain informed consent from each subject in accordance with Subject Information and Informed Consent.

A subject that qualified for an earlier cohort (but did not enroll) may be used in a subsequent cohort with no required re-screening if the Day 0 laboratory results meet the eligibility criteria.

The following procedures will be completed:

- Complete medical history.
- Complete history of all prescription or nonprescription drugs, and dietary supplements taken within 28 days prior to the planned first dose.
- Complete history of drug, alcohol and tobacco use.
- Physical examination including height and weight, blood pressure [BP] and pulse rate. The screening physical exam may be performed on Day 0, Period 1.
- Obtain single 12-lead electrocardiogram (ECG).

090177e180ac49ac\1.0\Approved\07-May-2009 14:41

- Following at least a 4-hour fast, blood and urine specimens for safety laboratory tests will be collected, in addition to the following:
    - Urine drug screen;
    - Urine cotinine;
    - Serum FSH concentrations for 45 to 55 year old post-menopausal females, defined as being amenorrheic for at least 2 years.

To prepare for study participation, subjects will be instructed on the use of Life Style Guidelines and Concomitant Medications sections.

Subjects previously screened for another study may participate in this provided they meet the subject selection criteria. Screening data from the previous study will be considered sufficient to satisfy the requirements of this study. Procedures required by this protocol will only be done if they were not performed for the previous study. All screening data must be obtained within 28 days prior to administration of study medication, as stipulated above.

6.2. Study Period

For the study period described below, where multiple procedures are scheduled at the same time point(s) relative to dosing, the following chronology of events should be adhered to, where possible.

- ECGs: obtain prior to vital signs and as close as possible to scheduled time, but prior to blood specimen collection.
- Blood pressure/pulse rate: obtain as close as possible to scheduled time, but prior to blood specimen collection.
- Pharmacokinetic blood specimens: obtain at scheduled time.
- Other procedures: all other procedures should be obtained as close as possible to the scheduled time, but may be obtained before or after blood specimen collection.

6.2.1. Day 0

Subjects will be admitted to the Clinical Research Unit the day prior to Day 1 dosing and will be required to stay in the CRU for 3 to 4 days. The following activities will be completed.

- Complete history of all prescription or nonprescription drugs, and dietary supplements taken within 28 days prior to dosing.
- Complete history of drug, alcohol and tobacco use.

090177e180ac49ac\1.0\Approved\07-May-2009 14:41

- Physical examination – A full physical screening exam may be performed at either Screening or on Day 0 (period 1 only) at the discretion of the CRU staff. A limited physical exam will be performed on Day 0 for Periods 2 and 3.

- Following at least a 4-hour fast, blood and urine specimens for safety laboratory tests will be collected, in addition to the following:

  - Urine drug screen.

  - Urine cotinine (Period 1 only).

Subjects will begin fasting at least 8 hours prior to dosing on Day 1 (except for Cohort 2, Period 4, see Section 4.4.1).

6.2.2. Day 1

Prior to dosing, the following activities will be completed:

- Initiate continuous cardiac monitoring at least 2 hours prior to dosing for Period 1 only (see Assessments; Cardiac Monitoring Section). This continuous cardiac monitoring may also be performed anytime after admission, but prior to dosing.

- Assess baseline symptoms/adverse events.

- Obtain triplicate 12-lead ECG measurements (see Section 7.2.3). May insert intravenous catheter for collection of blood samples.

- May insert intravenous catheter for collection of blood samples.

- Assess single supine blood pressure and pulse rate (see Section 7.2.2).

- Collect a blood sample for pharmacokinetic (PK) analysis (see Section 7.3.1).

- Collect whole blood for bactericidal activity (Cohort 1, Period 3 only and Cohort 2, Periods 1, 2 and 3 only, see Section 7.4.1).

- Cohort 2, Period 4 only- subjects should consume high calorie, high fat meal prior to dosing (see Section 4.4.1).

- Administer the study medication as described in the Study Treatments Section and, more specifically, in Administration Section (see Section 5.3.3).

After dosing, the following activities will be completed:

- Obtain triplicate 12-lead ECG measurements at approximately 1, 2, 3, 4, 8, and 12 hours after dosing.

090177e180ac49ac\1.0\Approved\07-May-2009 14:41

- Assess single supine blood pressure and pulse rate at approximately 1, 2, 3, 4, 8, and 12 hours after dosing.
- Collect blood samples for pharmacokinetic analysis at 1, 2, 3, 4, 8, 12 and 16 hours after dosing.
- Collect whole blood for bactericidal activity at 2, 3, 6, 12 hours after dosing. (Cohort 1, Period 3 only and Cohort 2, Periods 1, 2, and 3, see Section 7.4.1 for details).
- Maintain continuous cardiac monitoring for 8 hours (or longer if considered clinically necessary by the investigator) following dose administration (see Section 7.2.4).
- Assess symptoms by spontaneous reporting of adverse events and by asking the subjects to respond to a non-leading question such as “How do you feel?” at 1, 2, 4, 8, 12 and 16 hours after dosing.

6.2.3. Day 2

The following activities will be completed:

- Obtain triplicate 12-lead ECG measurements approximately 24 hours and 36 hours after dosing on Day 1.
- Assess single supine blood pressure and pulse rate approximately 24 and 36 hours after dosing on Day 1.
- Collect blood samples for pharmacokinetic analysis at 24 and 36 hours after dosing on Day 1.
- Collect whole blood for bactericidal activity at 24 hours after dosing on Day 1 (Cohort 1, Period 3 only and Cohort 2, Periods 1, 2, and 3, see Section 7.4.1 for details).
- Collect blood and urine samples for safety laboratory tests approximately 24 hours after dosing on Day 1.
- Assess symptoms by spontaneous reporting of adverse events and by asking the subjects to respond to a non-leading question such as “How do you feel?” at 24 hours and 36 after dosing.

6.2.4. Day 3

The following activities will be completed:

- Limited physical exam (Period 3 only for Cohort 1 and Period 4 only for Cohort 2 when not collecting a 72 hour pharmacokinetic analysis sample).

090177e180ac49ac\1.0\Approved\07-May-2009 14:41

- Obtain triplicate 12-lead ECG measurements approximately 48 hours after dosing on Day 1 (when not collecting a 72 hour pharmacokinetic analysis sample).
- Assess single supine blood pressure and pulse rate approximately 48 hours after dosing on Day 1.
- Collect blood and urine samples for safety laboratory tests approximately 48 hours after dosing on Day 1 (when not collecting a 72 hour pharmacokinetic analysis sample).
- Collect blood samples for pharmacokinetic analysis at 48 hours after dosing on Day 1.
- Assess symptoms by spontaneous reporting of adverse events and by asking the subjects to respond to a non-leading question such as “How do you feel?” at 48 hours after dosing.
- Discharge from the Clinical Research Unit (when not collecting a 72 hour pharmacokinetic analysis sample).

6.2.5. Day 4

The following activities will be completed:

- Limited physical exam (Period 3 only for Cohort 1 and Period 4 only for Cohort 2).
- Obtain triplicate 12-lead ECG measurements approximately 72 hours after dosing on Day 1.
- Assess single supine blood pressure and pulse rate approximately 72 hours after dosing on Day 1.
- Collect blood and urine samples for safety laboratory tests approximately 72 hours after dosing on Day 1.
- Collect blood samples for pharmacokinetic analysis at 72 hours after dosing on Day 1 (Cohort 1, Period 2 and 3, Cohort 2, Period 1, 2, 3, 4).
- Assess symptoms by spontaneous reporting of adverse events and by asking the subjects to respond to a non-leading question such as “How do you feel?” at 72 hours after dosing.
- Discharge from the Clinical Research Unit.

There will be an interval of at least 7 days between study periods (ie, administration of subsequent doses of study medication will not occur until at least 7 days after the previous

090177e180ac49ac\1.0\Approved\07-May-2009 14:41

dose of study medication). All study periods will be conducted identically as described above.

If a subject has any clinically significant, study-related abnormalities at the conclusion of a scheduled inpatient portion of the study, the Pfizer medical monitor (or designated representative) should be notified and the subject should be asked to remain in the Clinical Research Unit until such abnormalities resolve. If the subject is unable or unwilling to remain in the Clinical Research Unit, the Pfizer medical monitor (or designated representative) should be so notified, and the investigator should make every effort to arrange follow-up evaluations at appropriate intervals to document the course of the abnormalities.

6.3. Follow-up Visit

Subjects will return to the Clinical Research Unit 7 to 14 days following the last dose of study medication for a follow-up visit. At this visit, the following procedures will be completed:

- Obtain information regarding the use of concomitant medication and the occurrence of adverse events.
- Assess supine blood pressure and pulse rate.
- Obtain 12-lead ECG measurement.
- Collect blood and urine samples for safety laboratory tests.
- Conduct physical examination only if an AE is unresolved, or at the discretion on the investigator.

6.4. Post-Study Subject Interview

Information related to a subject's personal experience in this study might be helpful for the planning and execution of subsequent studies. Therefore, the sponsor (or designate) may, with the agreement of the investigator, conduct voluntary interviews with all or some of the study subjects. The investigator or investigator site personnel will initially contact subjects to assess their interest in participating in the interview and to schedule a convenient time. Interviews will be conducted at the investigator site and will last approximately 30 to 60 minutes. Interview information will not be included in the study database and will not be reported.

6.5. Subject Withdrawal

Subjects may withdraw from the study at any time at their own request, or they may be withdrawn at any time at the discretion of the investigator or sponsor for safety, behavioral, or administrative reasons. If a subject does not return for a scheduled visit, every effort should be made to contact the subject. In any circumstance, every effort should be made to document subject outcome, if possible. The investigator should inquire about the reason for

090177e180ac49ac\1.0\Approved\07-May-2009 14:41

withdrawal, request the subject to return for a final visit, if applicable, and follow-up with the subject regarding any unresolved adverse events.

In the event that a subject is withdrawn from the study prematurely, regardless of the reason for withdrawal, subjects will receive the following prior to discharge:

- Limited physical examination.
- Single 12-lead ECG.
- Single blood pressure and pulse rate measurements.
- Blood and urine specimens for safety laboratory tests.
- Blood sample for pharmacokinetic analysis.
- Assess symptoms by spontaneous reporting of adverse events and by asking the subjects to respond to a non-leading question such as "How do you feel?"
- Obtain information regarding the use of concomitant medication.

If the subject withdraws from the study and also withdraws consent for disclosure of future information, no further evaluations should be performed and no additional data should be collected. The sponsor may retain and continue to use any data collected before such withdrawal of consent.

7. ASSESSMENTS

7.1. Blood Volume

Total blood sampling volume for the individual subjects is approximately 272 mL. The actual times of blood sampling may change. Additional blood samples may be taken at times specified by Pfizer, provided the total volume taken during the study does not exceed 550 mL and the IRB/IEC is notified of the blood collection.

090177e180ac49ac\1.0\Approved\07-May-2009 14:41

Table 8. Blood Volume

Sample Type	Sample Volume (mL)	Number of Sampling Times			Total Volume (mL)
		Screening	Study Period	Follow-Up	
Safety Labs	7	1	9 (1); 12 (2)	1	77 (1); 98 (2)
PK	4		35 (1); 48 (2)		140 (1); 192 (2)
Bactericidal Activity*	3		6(1); 18(2)		18(1); 54(2)
TOTAL**					235 (1); 344 (2)

* Cohort 1, Period 3 only; Cohort 2, Period 1, 2 and 3 only.
**This total volume does not include discarded blood from predraws used to remove fluid from flushed catheters.

7.2. Safety

7.2.1. Laboratory

The following safety laboratory tests will be performed at times defined in the Study Period section of this protocol.

Table 9. Safety Laboratory Assessments

Safety Laboratory

Hematology	Chemistry	Urinalysis	Other
Hemoglobin<br>Hematocrit<br>RBC count<br>Reticulocyte count<br>Platelet count<br>WBC count<br>Total neutrophils (Abs)<br>Eosinophils (Abs)<br>Monocytes (Abs)<br>Basophils (Abs)<br>Lymphocytes (Abs)	BUN and Creatinine<br>Glucose (fasting)<br>Calcium<br>Sodium<br>Potassium<br>Chloride<br>Total CO2 (Bicarbonate)<br>AST, ALT<br>GGT<br>Total Bilirubin<br>Alkaline phosphatase<br>Uric acid<br>Albumin<br>Total protein	pH<br>Glucose (qual)<br>Protein (qual)<br>Blood (qual)<br>Ketones<br>Microscopy[a]	Urine drug screen[b]<br>Urine cotinine[c]<br>FSH (if applicable)[d]

[a] Only if urine dipstick is positive for blood or protein
[b] Screening and Day 0 of each Period
[c] Screening and Day 0 of Period 1 only
[d] Screening only

- Urine drug testing will be done at Screening and Day 0 of each Period. Minimum requirement for drug screening includes: cocaine, THC, opiates, benzodiazepines and amphetamines.

- Cotinine testing will be done at Screening and Day 0 in Period 1 only.

090177e180ac49ac\1.0\Approved\07-May-2009 14:41

- Serum FSH concentrations for 45 to 55 year old post-menopausal females to confirm status at screening only.

- Safety laboratory tests from Day 0 must have no clinically significant findings, as judged by the investigator, in order for a subject to be dosed on Day 1.

7.2.2. Blood Pressure and Pulse Rate

Single supine blood pressure and pulse rate will be measured at times specified in Section 6 of this protocol. Additional collection times, or changes to collection times of blood pressure and pulse rate will be permitted, as necessary, to ensure appropriate collection of safety data.

Supine blood pressure will be measured with the subject's arm supported at the level of the heart, and recorded to the nearest mm Hg. The same arm (preferably the dominant arm) will be used throughout the study. All scheduled BP/PR assessments should be performed after the subject has rested quietly for at least 5 minutes in a supine position.

The same size blood pressure cuff, which has been properly sized and calibrated, will be used to measure blood pressure each time. The use of automated devices for measuring BP and pulse rate are acceptable, although, when done manually, pulse rate will be measured in the brachial/radial artery for at least 30 seconds. When the timing of these measurements coincides with a blood collection, blood pressure and pulse rate should be obtained prior to the nominal time of the blood collection.

7.2.3. Electrocardiogram (ECG)

ECGs should be collected at times specified in the Study Period section of this protocol.

All scheduled ECGs should be performed after the subject has rested quietly for at least 10 minutes in a supine position. A single 12-lead ECG will be obtained on all subjects at screening and follow-up.

Triplicate 12-lead ECGs will be obtained approximately 2-4 minutes apart; and the average of the triplicate ECG measurements collected predose on Day 1 of each Period will serve as each subject's baseline QTc value. When the timing of these measurements coincides with a blood collection, the ECG should be obtained prior to the nominal time of the blood collection, blood pressure and pulse rate.

To ensure safety of the subjects, a qualified individual at the investigator site will make comparisons to the predose Day 1 baseline. If the mean QTc of any triplicate measurement is >45 msec from the time-controlled baseline; or an absolute QTc value is ≥500 msec for any scheduled ECG, a single ECG will be repeated at least hourly until QTc values from 2 successive ECGs fall below the threshold value that triggered the repeat measurement.

In some cases, it may be appropriate to repeat abnormal ECGs to rule out improper lead placement as contributing to the ECG abnormality. It is important that leads are placed in the same positions each time in order to achieve precise ECG recordings. If a machine-read QTc

090177e180ac49ac\1.0\Approved\07-May-2009 14:41

value is prolonged, as defined above, repeat measurements may not be necessary if a qualified physician's interpretation determines that the QTc values are in the acceptable range.

7.2.4. Continuous Cardiac Monitoring by Telemetry

All abnormal rhythms will be recorded and reviewed by the study physician for the presence of rhythms of potential clinical concern. The time, duration, and description of the event will be recorded in the CRF. In addition, a printed record of the tracing(s) of the clinically significant rhythm(s) will be made and retained with other source documents.

Telemetry should be collected using a centralized system that also allows for the storage and advanced analysis of all recorded data, in order to preserve the important events for future evaluations. Holter monitoring should not be used in parallel with continuous telemetry, unless it is the only means of data storage available at the study site, or verifiable arrhythmia quantification is required. To establish a baseline, telemetry should be recorded for at least 2 hours before dosing (Period 1 only) and continue through 8 hours postdose for all cohorts.

Telemetry may be stopped within a reasonably short period of time prior to dosing, in order to avoid interference with study operations conducted immediately before dosing. However, it is expected that the telemetry leads will be in place and the system connected prior to dosing.

7.3. Pharmacokinetics

7.3.1. Plasma for Analysis of PNU-100480 and Metabolites

During all study periods, blood samples (4 mL) to provide a minimum of 2 mL plasma for pharmacokinetic analysis will be collected into appropriately labeled tubes containing K2 EDTA at times specified in the Schedule of Activities.

The actual times may change but the number of samples will remain the same. All efforts will be made to obtain the pharmacokinetic samples at the exact nominal time relative to dosing. However, samples obtained within 10% of the nominal time (eg, within 6 minutes of a 60 minute sample) from dosing will not be captured as a protocol deviation, as long as the exact time of the sample collection is noted on the source document and data collection tool (eg, CRF).

The whole blood will remain at room temperature until clotted (approximately 30 minutes) then samples will be centrifuged at approximately 1700 g for about 10 minutes at 4°C. The serum will be transferred into appropriately labeled screw-capped polypropylene tubes at approximately -20°C within 1 hour of collection.

Samples will be analyzed using a validated analytical method in compliance with Pfizer standard operating procedures.

090177e180ac49ac\1.0\Approved\07-May-2009 14:41

As part of understanding the pharmacokinetics of the study drug and its metabolites, samples may be used for metabolite identification and/or evaluation of the bioanalytical method. These data will be used for internal exploratory purposes and will not be included in the clinical report.

7.3.2. Shipment of Pharmacokinetic Samples

The shipment address and assay lab contact information will be provided to the investigator site prior to initiation of the study.

7.4. Pharmacodynamics (Whole Blood Bactericidal Activity)

7.4.1. Whole Blood for Bactericidal Activity Sample Collection

During specified study periods, blood samples of a minimum of 3 mL for assessment of whole blood bactericidal activity will be collected into appropriately labeled sodium heparin containing tubes.

- Batch 1 samples: Predose and 2, 3, 6 hours after dosing.
- Batch 2 samples: 12 and 24 hours after dosing.

The whole blood bactericidal assay requires fresh whole blood samples, and the dosing and whole blood sampling must therefore be coordinated with the availability of the assay facility.

All efforts will be made to obtain the whole blood samples at the exact nominal time relative to dosing. The actual times may be modified to accommodate the availability of the assay facility. It is anticipated that the availability of the facility may on certain occasions preclude collection of Batch 2. In such cases, Batch 2 will be omitted. In all cases, the number of samples will remain the same or fewer.

7.4.2. Bactericidal Activity Sample Shipment

Samples are to remain at room temperature until they are transported to the assay laboratory approximately 90 minutes after collection of the last batched specimen. Batch 1 will be transported on Day 1; Batch 2 will be transported on Day 2. Laboratory contact information will be provided to the site prior to study start.

7.4.3. Analysis of Whole Blood for Bactericidal Activity

Laboratory methods and data reporting for whole blood bactericidal activity will be provided to the site prior to study start.

090177e180ac49ac\1.0\Approved\07-May-2009 14:41

090177e180ac49ac\1.0\Approved\07-May-2009 14:41

8. ADVERSE EVENT REPORTING

8.1. Adverse Events

All observed or volunteered adverse events regardless of treatment group or suspected causal relationship to the investigational product(s) will be reported as described in the following sections.

For all adverse events, the investigator must pursue and obtain information adequate both to determine the outcome of the adverse event and to assess whether it meets the criteria for classification as a serious adverse event requiring immediate notification to Pfizer or its designated representative. For all adverse events, sufficient information should be obtained by the investigator to determine the causality of the adverse event. The investigator is required to assess causality. For adverse events with a causal relationship to the investigational product, follow-up by the investigator is required until the event or its sequelae resolve or stabilize at a level acceptable to the investigator, and Pfizer concurs with that assessment.

8.2. Reporting Period

For serious adverse events, the reporting period to Pfizer or its designated representative begins from the time that the subject provides informed consent, which is obtained prior to the subject's participation in the study, ie, prior to undergoing any study-related procedure and/or receiving investigational product, through and including 28 calendar days after the last administration of the investigational product. Any serious adverse event occurring any time after the reporting period must be promptly reported if a causal relationship to investigational product is suspected.

- Adverse events (serious and non-serious) should be recorded on the CRF from the time the subject has taken at least one dose of study treatment through last subject visit.

8.3. Definition of an Adverse Event

An adverse event is any untoward medical occurrence in a clinical investigation subject administered a product or medical device; the event need not necessarily have a causal relationship with the treatment or usage. Examples of adverse events include but are not limited to:

- Abnormal test findings;
- Clinically significant symptoms and signs;
- Changes in physical examination findings;
- Hypersensitivity;

Additionally, they may include the signs or symptoms resulting from:

- Drug overdose;
- Drug withdrawal;
- Drug abuse;
- Drug misuse;
- Drug interactions;
- Drug dependency;
- Extravasation;
- Exposure in Utero.

8.4. Abnormal Test Findings

The criteria for determining whether an abnormal objective test finding should be reported as an adverse event are as follows:

- Test result is associated with accompanying symptoms, and/or
- Test result requires additional diagnostic testing or medical/surgical intervention, and/or
- Test result leads to a change in study dosing or discontinuation from the study, significant additional concomitant drug treatment, or other therapy, and/or
- Test result is considered to be an adverse event by the investigator or sponsor.

Merely repeating an abnormal test, in the absence of any of the above conditions, does not constitute an adverse event. Any abnormal test result that is determined to be an error does not require reporting as an adverse event.

8.5. Serious Adverse Events

A serious adverse event or serious adverse drug reaction is any untoward medical occurrence at any dose that:

- Results in death;
- Is life-threatening (immediate risk of death);
- Requires inpatient hospitalization or prolongation of existing hospitalization;

090177e180ac49ac\1.0\Approved\07-May-2009 14:41

- Results in persistent or significant disability/incapacity;
- Results in congenital anomaly/birth defect.

Medical and scientific judgment should be exercised in determining whether an event is an important medical event. An important medical event may not be immediately life-threatening and/or result in death or hospitalization. However, if it is determined that the event may jeopardize the subject and/or may require intervention to prevent one of the other adverse event outcomes, the important medical event should be reported as serious.

Examples of such events are intensive treatment in an emergency room or at home for allergic bronchospasm; blood dyscrasias or convulsions that do not result in hospitalization; or development of drug dependency or drug abuse.

8.6. Hospitalization

Adverse events reported from studies associated with hospitalization or prolongation of hospitalization, are considered serious. Any initial admission (even if less than 24 hours) to a healthcare facility meets these criteria. Admission also includes transfer within the hospital to an acute/intensive care unit (eg, from the psychiatric wing to a medical floor, medical floor to a coronary care unit, neurological floor to a tuberculosis unit).

Hospitalization does not include the following:

- Rehabilitation facilities;
- Hospice facilities;
- Respite care (eg, caregiver relief);
- Skilled nursing facilities;
- Nursing homes;
- Routine emergency room admissions;
- Same day surgeries (as outpatient/same day/ambulatory procedures).

Hospitalization or prolongation of hospitalization in the absence of a precipitating, clinical adverse event is not in itself a serious adverse event. Examples include:

- Admission for treatment of a preexisting condition not associated with the development of a new adverse event or with a worsening of the preexisting condition (eg, for work-up of persistent pre-treatment lab abnormality);
- Social admission (eg, subject has no place to sleep);

090177e180ac49ac\1.0\Approved\07-May-2009 14:41

- Administrative admission (eg, for yearly physical exam);
- Protocol-specified admission during a study (eg, for a procedure required by the study protocol);
- Optional admission not associated with a precipitating clinical adverse event (eg, for elective cosmetic surgery);
- Pre-planned treatments or surgical procedures should be noted in the baseline documentation for the entire protocol and/or for the individual subject.

Diagnostic and therapeutic non-invasive and invasive procedures, such as surgery, should not be reported as adverse events. However, the medical condition for which the procedure was performed should be reported if it meets the definition of an adverse event. For example, an acute appendicitis that begins during the adverse event reporting period should be reported as the adverse event, and the resulting appendectomy should be recorded as treatment of the adverse event.

8.7. Severity Assessment

If required on the adverse event case report forms, the investigator will use the adjectives MILD, MODERATE, or SEVERE to describe the maximum intensity of the adverse event. For purposes of consistency, these intensity grades are defined as follows:	
MILD	Does not interfere with subject's usual function.
MODERATE	Interferes to some extent with subject's usual function.
SEVERE	Interferes significantly with subject's usual function.

Note the distinction between the severity and the seriousness of an adverse event. A severe event is not necessarily a serious event. For example, a headache may be severe (interferes significantly with subject's usual function) but would not be classified as serious unless it met one of the criteria for serious adverse events, listed above.

8.8. Causality Assessment

The investigator's assessment of causality must be provided for all adverse events (serious and non-serious); the investigator must record the causal relationship in the CRF, as appropriate, and report such an assessment in accordance with the serious adverse reporting requirements if applicable. An investigator's causality assessment is the determination of whether there exists a reasonable possibility that the investigational product caused or contributed to an adverse event. If the investigator's final determination of causality is unknown and the investigator does not know whether or not investigational product caused the event, then the event will be handled as "related to investigational product" for reporting purposes, as defined by the Sponsor (see Section on Reporting Requirements). If the investigator's causality assessment is "unknown but not related to investigational product", this should be clearly documented on study records.

090177e180ac49ac\1.0\Approved\07-May-2009 14:41

In addition, if the investigator determines a serious adverse event is associated with study procedures, the investigator must record this causal relationship in the source documents and CRF, as appropriate, and report such an assessment in accordance with the serious adverse event reporting requirements, if applicable.

8.9. Exposure In Utero

For investigational products and for marketed products, an exposure in-utero (EIU) occurs if:

1. A female becomes, or is found to be, pregnant either while receiving or having been directly exposed to (eg, environmental exposure) the investigational product, or the female becomes, or is found to be, pregnant after discontinuing and/or being directly exposed to the investigational product (maternal exposure);

2. A male has been exposed, either due to treatment or environmental, to the investigational product prior to or around the time of conception and/or is exposed during his partner's pregnancy (paternal exposure).

If any study subject or study subject's partner becomes or is found to be pregnant during the study subject's treatment with the investigational product, the investigator must submit this information to Pfizer on an Exposure in Utero Form. In addition, the investigator must submit information regarding environmental exposure to a Pfizer product in a pregnant woman (eg, a nurse reports that she is pregnant and has been exposed to a cytotoxic product by inhalation or spillage) using the Exposure in Utero Form. This must be done irrespective of whether an adverse event has occurred and within 24 hours of awareness of the pregnancy. The information submitted should include the anticipated date of delivery (see below for information related to induced termination of pregnancy).

Follow-up is conducted to obtain pregnancy outcome information on all Exposure in Utero reports with an unknown outcome. The investigator will follow the pregnancy until completion or until pregnancy termination (ie, induced abortion) and then notify Pfizer of the outcome. The investigator will provide this information as a follow up to the initial Exposure in Utero Form. The reason(s) for an induced abortion should be specified. An EIU report is not created when an ectopic pregnancy report is received since this pregnancy is not usually viable. Rather, a serious adverse event case is created with the event of ectopic pregnancy.

If the outcome of the pregnancy meets the criteria for immediate classification as a serious adverse event (ie, spontaneous abortion, stillbirth, neonatal death, or congenital anomaly [including that in an aborted fetus, stillbirth or neonatal death]), the investigator should follow the procedures for reporting serious adverse events.

In the case of a live birth, the "normality" of the newborn can be assessed at the time of birth (ie, no minimum follow-up period of a presumably normal infant is required before an Exposure in Utero Form can be completed). The "normality" of an aborted fetus can be assessed by gross visual inspection, unless pre-abortion test findings are suggestive of a congenital anomaly.

090177e180ac49ac\1.0\Approved\07-May-2009 14:41

Additional information about pregnancy outcomes that are classified as serious adverse events follows:

- "Spontaneous abortion" includes miscarriage and missed abortion.
- All neonatal deaths that occur within 1 month of birth should be reported, without regard to causality, as serious adverse events. In addition, any infant death after 1 month that the investigator assesses as possibly related to the exposure in utero to the investigational medication should be reported.

Additional information regarding the exposure in utero may be requested by the investigator. Further follow-up of birth outcomes will be handled on a case-by-case basis (eg, follow-up on preterm infants to identify developmental delays). In the case of paternal exposure, the investigator must obtain permission from the subject's partner in order to conduct any follow-up or collect any information.

8.10. Withdrawal Due to Adverse Events (See Also Section on Subject Withdrawal)

Withdrawal due to adverse event should be distinguished from withdrawal due to insufficient response, according to the definition of adverse event noted earlier, and recorded on the appropriate adverse event CRF page.

When a subject withdraws due to a serious adverse event, the serious adverse event must be reported in accordance with the reporting requirements defined below.

8.11. Eliciting Adverse Event Information

The investigator is to report all directly observed adverse events and all adverse events spontaneously reported by the study subject. In addition, each study subject will be questioned about adverse events.

8.12. Reporting Requirements

Each adverse event is to be assessed to determine if it meets the criteria for serious adverse events. If a serious adverse event occurs, expedited reporting will follow local and international regulations, as appropriate.

8.12.1. Serious Adverse Event Reporting Requirements

If a serious adverse event occurs, Pfizer is to be notified within 24 hours of awareness of the event by the investigator. In particular, if the serious adverse event is fatal or life-threatening, notification to Pfizer must be made immediately, irrespective of the extent of available adverse event information. This timeframe also applies to additional new information (follow-up) on previously forwarded serious adverse event reports as well as to the initial and follow-up reporting of Exposure in Utero cases.

090177e180ac49ac\1.0\Approved\07-May-2009 14:41

This timeframe of reporting within 24 hours also applies for the reporting of any medical device complaint.

In the rare event that the investigator does not become aware of the occurrence of a serious adverse event immediately (eg, if an outpatient study subject initially seeks treatment elsewhere), the investigator is to report the event within 24 hours after learning of it and document the time of his/her first awareness of the adverse event.

For all serious adverse events, the investigator is obligated to pursue and provide information to Pfizer in accordance with the timeframes for reporting specified above. In addition, an investigator may be requested by Pfizer to obtain specific additional follow-up information in an expedited fashion. This information may be more detailed than that captured on the adverse event case report form. In general, this will include a description of the adverse event in sufficient detail to allow for a complete medical assessment of the case and independent determination of possible causality. Information on other possible causes of the event, such as concomitant medications and illnesses must be provided. In the case of a subject death, a summary of available autopsy findings must be submitted as soon as possible to Pfizer or its designated representative.

8.12.2. Non-Serious Adverse Event Reporting Requirements

All adverse events will be reported on the adverse event page(s) of the CRF. It should be noted that the form for collection of serious adverse event information is not the same as the adverse event CRF. Where the same data are collected, the forms must be completed in a consistent manner. For example, the same adverse event term should be used on both forms. Adverse events should be reported using concise medical terminology on the CRFs as well as on the form for collection of serious adverse event information.

8.12.3. Sponsor Reporting Requirements to Regulatory Authorities

Adverse events reporting, including suspected serious unexpected adverse reactions, will be carried out in accordance with applicable local regulations.

9. DATA ANALYSIS/STATISTICAL METHODS

Detailed methodology for summary and statistical analyses of the data collected in this trial will be documented in a Statistical Analysis Plan, which will be dated and maintained by the sponsor. This document may modify the plans outlined in the protocol; however, any major modifications of the primary endpoint definition and/or its analysis will also be reflected in a protocol amendment.

9.1. Sample Size Determination

A total sample size of up to 18 healthy subjects has been chosen based on the need to minimize first exposure to humans of a new chemical entity and the requirement to provide adequate safety and toleration information at each dose level. Subjects who withdraw prior

090177e180ac49ac\1.0\Approved\07-May-2009 14:41

to the completion of the study may be replaced at the discretion of the Sponsor. No formal inferential statistics will be applied to the pharmacokinetic, pharmacodynamic or safety data. These will be summarized descriptively.

9.2. Efficacy Analysis

Efficacy analysis is not applicable to this trial.

9.3. Pharmacokinetic Analysis

9.3.1. Analysis Populations

All enrolled subjects with pharmacokinetic parameter(s) of interest for at least one period of the study will be included in the statistical analysis. Analysis sets may contain different numbers of subjects for different pharmacokinetic parameters based on availability of data. Individual and mean plasma PNU-100480, PNU-101603 and PNU-101244 concentration-time data will be tabulated and plotted as a function of dose.

9.3.2. Derivation of Pharmacokinetic Parameters

The pharmacokinetic parameters for PNU-100480, PNU-101603 and PNU-101244 will be determined for each dose of PNU-100480 using non-compartmental methods, as data permit: maximum observed plasma concentration (C_{max}), the time to C_{max} (Tmax), the area under the concentration-time curve from time 0 to the time of the last quantifiable concentration (AUC_{last}), the area under the concentration-time curve estimated from time 0 to 24 hours (AUC_{24}) and infinity (AUC_{inf}) and the terminal phase half-life (t1/2). Also, the apparent oral clearance (CL/F) and the apparent volume of distribution (Vz/F) will be calculated for PNU-100480. PK parameters will be derived from the concentration-time profiles as follows:

090177e180ac49ac\1.0\Approved\07-May-2009 14:41

090177e180ac49ac\1.0\Approved\07-May-2009 14:41

Table 10. Pharmacokinetic Parameters

Parameter	Definition	Method of Determination
AUC_{last}	Area under the plasma concentration-time profile from time zero to the time of the last quantifiable concentration (C_{last})	Linear/Log trapezoidal method
AUC_{inf}	Area under the plasma concentration-time profile from time zero extrapolated to infinite time	AUC_{last} + (Clast*/kel), where C_{last}* is the predicted plasma concentration at the last quantifiable time point estimated from the log-linear regression analysis.
AUC_{24}	Area under the plasma concentration-time profile from time zero extrapolated to 24 hours	Linear/Log trapezoidal method
C_{max}	Maximum plasma concentration	Observed directly from data
T_{max}	Time for C_{max}	Observed directly from data as time of first occurrence
t1/2	Terminal elimination half-life	Loge(2)/kel, where kel is the terminal phase rate constant calculated by a linear regression of the log-linear concentration-time curve. Only those data points judged to describe the terminal log-linear decline will be used in the regression.
CL/F	Apparent clearance	Dose/ AUC_{inf}
Vz/F	Apparent volume of distribution	Dose/ (AUC_{inf} . kel)

Actual PK sampling times will be used in the derivation of PK parameters. The relationship between relevant ECG parameters (eg, QRS and QT interval) and PNU-100480, PNU-101603 and PNU-101244 plasma concentrations will be explored graphically. If appropriate structural pharmacodynamic models may also be utilized to characterize these relationships using PK/PD approach.

9.3.3. Statistical Methods

No formal inferential statistics will be applied to the pharmacokinetic data.

The plasma PK parameters AUC_{inf}, AUC_{24}, Cmax, AUC_{last}, Tmax and $t_{1/2}$ for PNU-100480 and its metabolite will be summarized descriptively by analyte, cohort, fasted/fed condition, and dose. Also, CL/F and Vz/F for PNU-100480 will be summarized descriptively by dose and fasted/fed condition. Plasma concentrations will be listed and summarized descriptively by analyte, cohort, dose, fasted/fed condition, and nominal PK sampling time. Individual

subject and median profiles of the plasma concentration-time data will be plotted by dose using actual and nominal times respectively for each analyte. Median profiles will be presented on both linear-linear and log-linear scales.

Dose normalized (to a 1 mg) AUC_{inf}, AUC_{last} and C_{max} for PNU-100480 and its metabolite will be plotted against dose (using a logarithmic scale) and will include individual subject values and the geometric means for each dose. These plots will be used to help understand the relationship between the plasma PK parameters and dose.

9.4. Pharmacodynamic Analysis

9.4.1. Whole Blood Bactericidal Activity (WBA)

The absolute values and changes from baseline for WBA will be summarized descriptively (N, mean, median, minimum, maximum, standard deviation) by dose and time-points. These summaries will be calculated for each dose level cohort.

The relationship between WBA and PNU-100480 (or its metabolite) concentration may be explored graphically or statistically.

9.5. Safety Analysis

The safety analysis population is defined as all enrolled subjects who receive at least 1 dose of study medication.

Adverse events, ECGs, blood pressure, pulse rate, continuous cardiac monitoring, and safety laboratory data will be reviewed and summarized on an ongoing basis during the study to evaluate the safety of subjects. Any clinical laboratory, ECG, BP, and PR abnormalities of potential clinical concern as defined in Appendix 1 will be described. Safety data will be presented in tabular and/or graphical format and summarized descriptively, where appropriate.

Medical history and physical exam information collected during the course of the study will not be captured for inclusion into the study database, unless otherwise noted. However, any untoward findings identified on physical exams conducted after the administration of the first dose of study medication will be captured as an adverse event, if those findings meet the definition of an adverse event. Data collected at screening that is used for inclusion/exclusion criteria, such as laboratory data, ECGs and vital signs will be considered source data, and will not be captured for inclusion into the study database, unless otherwise noted. Demographic data collected at screening will be included in the study database.

Routinely collected laboratory safety data that are not required by the protocol will be considered source data, and suppressed at the point of mapping data from PIMS into PIMS data extraction views. However, unexpected data collected with specific medical intent (ie, the workup of an adverse event, etc.) that is not required by the protocol, will not be suppressed at the point of extraction from PIMS.

090177e180ac49ac\1.0\Approved\07-May-2009 14:41

Key data from the telemetry or Holter summary reports, including PVC frequency, PAC frequency, supraventricular couplets, ventricular couplets, occurrence, duration and morphology of ventricular tachycardia, and occurrence and type of AV block, will be captured electronically and transferred to a central database. The primary purpose of this collection is to allow the construction of a database summarizing the frequency of events in drug treated subjects and, more importantly, establish the background event rate in placebo treated subjects. The background event rate will permit the proper perspective to be placed on observations arising from future clinical studies. Cross-consistency between the database and the AE forms will not be checked. The database will be housed in Oracle Clinical under protocol NRA9990006, "Cardiac Telemetry Dataset Analysis," for the initial year that this guidance is in effect.

9.5.1. Electrocardiogram (ECG) Analysis

9.5.1.1. Derivation of Electrocardiogram Parameters

The QT, heart rate, QTcB, QTcF, PR, RR, and QRS will be recorded at each assessment time indicated in the Schedule of Activities. If QTcF is not supplied then it will be derived using Fridericia's (QTcF) heart rate correction formula. If QTcB is not supplied then it will be calculated using Bazett's heart rate correction formula.

The average of the triplicate readings collected at each assessment time will be calculated prior to analyzing each ECG parameter. The average of the triplicate ECG measurements collected at predose on Day 1 will serve as each subject's baseline values. The maximum absolute value (postdose) and the maximum increase from baseline for QT, heart rate, QTcF, PR and QRS will be determined for each subject.

9.5.1.2. Statistical Methods

No formal inferential statistics will be applied to the ECG data.

Absolute values and changes from baseline in QT, heart rate, QTcF, PR and QRS will be summarized by cohort, treatment and time postdose. Maximum absolute values and maximum increase from baseline for QT, heart rate, QTcF, PR and QRS will be summarized by cohort and treatment.

In addition for baseline subtracted QT, heart rate and QTcF, the differences between each dose and placebo (dose – placebo) for each subject will be summarized and plotted (N, mean, 90% confidence interval) for each cohort, dose and time point (including baseline).

ECG endpoints and changes from baseline (QTcF, PR and QRS), over all measurements taken postdose, will also be summarized descriptively by cohort and treatment using categories as defined in the statistical analysis plan. Numbers and percentages of subjects meeting the categorical criteria will be provided and individual values listed in the study report. Listings of subjects with any single post-dose value ≥500 msec will also be produced for QTcF.

090177e180ac49ac\1.0\Approved\07-May-2009 14:41

Changes from baseline for QTcF will be plotted against PNU-100480 concentration.

9.5.2. Telemetry Analysis

Abnormal telemetry recordings will only be listed.

10. QUALITY CONTROL AND QUALITY ASSURANCE

During study conduct, Pfizer or its agent will conduct periodic monitoring visits to ensure that the protocol and GCPs are being followed. The monitors may review source documents to confirm that the data recorded on CRFs is accurate. For studies conducted outside of Pfizer Clinical Research Units, the investigator and institution will allow Pfizer monitors or its agents and appropriate regulatory authorities direct access to source documents to perform this verification.

The study site may be subject to review by the Institutional Review Board (IRB)/Independent Ethics Committee (IEC), and/or to quality assurance audits performed by Pfizer, or companies working with or on behalf of Pfizer, and/or to inspection by appropriate regulatory authorities.

For studies conducted outside of the Pfizer Clinical Research Units, it is important that the investigator(s) and their relevant personnel are available during the monitoring visits and possible audits or inspections and that sufficient time is devoted to the process.

11. DATA HANDLING AND RECORD KEEPING

11.1. Case Report Forms/Electronic Data Record

As used in this protocol, the term Case Report Form (CRF) should be understood to refer to either a paper form or an electronic data record or both, depending on the data collection method used in this study.

A CRF is required and should be completed for each included subject. The completed original CRFs are the sole property of Pfizer and should not be made available in any form to third parties, except for authorized representatives of Pfizer or appropriate regulatory authorities, without written permission from Pfizer.

The investigator has ultimate responsibility for the collection and reporting of all clinical, safety and laboratory data entered on the CRFs and any other data collection forms (source documents) and ensuring that they are accurate, authentic / original, attributable, complete, consistent, legible, timely (contemporaneous), enduring and available when required. The CRFs must be signed by the investigator or by an authorized staff member to attest that the data contained on the CRFs is true. Any corrections to entries made in the CRFs, source documents must be dated, initialed and explained (if necessary) and should not obscure the original entry."

090177e180ac49ac\1.0\Approved\07-May-2009 14:41

In most cases the source documents are the hospital or the physician's chart. In these cases, data collected on the CRFs must match those charts.

In some cases, the CRF may also serve as the source document. In these cases, a document should be available at the investigator's site as well as at Pfizer and clearly identify those data that will be recorded in the CRF, and for which the CRF will stand as the source document.

11.2. Record Retention

To enable evaluations and/or audits from regulatory authorities or Pfizer, the investigator agrees to keep records, including the identity of all participating subjects (sufficient information to link records, eg, CRFs and hospital records), all original signed informed consent forms, copies of all CRFs, serious adverse event forms, source documents, and detailed records of treatment disposition, and adequate documentation of relevant correspondence (eg, letters, meeting minutes, telephone calls reports). The records should be retained by the investigator according to ICH, local regulations, or as specified in the Clinical Study Agreement, whichever is longer.

If the investigator becomes unable for any reason to continue to retain study records for the required period (eg, retirement, relocation), Pfizer should be prospectively notified. The study records must be transferred to a designee acceptable to Pfizer, such as another investigator, another institution, or to Pfizer. The investigator must obtain Pfizer's written permission before disposing of any records, even if retention requirements have been met.

12. ETHICS

12.1. Institutional Review Board (IRB)/Independent Ethics Committee (IEC)

It is the responsibility of the investigator to have prospective approval of the study protocol, protocol amendments, informed consent forms, and other relevant documents, eg, recruitment advertisements, if applicable, from the IRB/IEC. All correspondence with the IRB/IEC should be retained in the Investigator File. Copies of IRB/IEC approvals should be forwarded to Pfizer.

The only circumstance in which an amendment may be initiated prior to IRB/IEC approval is where the change is necessary to eliminate apparent immediate hazards to the subjects. In that event, the investigator must notify the IRB/IEC and Pfizer in writing immediately after the implementation.

12.2. Ethical Conduct of the Study

The study will be conducted in accordance with legal and regulatory requirements, as well as the general principles set forth in the International Ethical Guidelines for Biomedical Research Involving Human Subjects (Council for International Organizations of Medical Sciences 2002), Guidelines for Good Clinical Practice (International Conference on Harmonization 1996), and the Declaration of Helsinki (World Medical Association 2008).

090177e180ac49ac\1.0\Approved\07-May-2009 14:41

If mandated by local law, compliance with local requirements may be inserted in this paragraph.

In addition, the study will be conducted in accordance with the protocol, the International Conference on Harmonisation guideline on Good Clinical Practice, and applicable local regulatory requirements and laws.

12.3. Subject Information and Consent

All parties will ensure protection of subject personal data and will not include subject names on any sponsor forms, reports, publications, or in any other disclosures, except where required by laws. In case of data transfer, Pfizer will maintain high standards of confidentiality and protection of subject personal data.

The informed consent form must be in compliance with ICH GCP, local regulatory requirements, and legal requirements.

The informed consent form used in this study, and any changes made during the course of the study, must be prospectively approved by both the IRB/IEC and Pfizer before use.

The investigator must ensure that each study subject, or his/her legally acceptable representative, is fully informed about the nature and objectives of the study and possible risks associated with participation. The investigator, or a person designated by the investigator, will obtain written informed consent from each subject or the subject's legally acceptable representative before any study-specific activity is performed. The investigator will retain the original of each subject's signed consent form.

12.4. Reporting of Safety Issues and Serious Breaches of the Protocol or ICH GCP

In the event of any prohibition or restriction imposed (ie, clinical hold) by an applicable Competent Authority in any area of the World, or if the investigator is aware of any new information which might influence the evaluation of the benefits and risks of the investigational product, Pfizer should be informed immediately.

In addition, the investigator will inform Pfizer immediately of any urgent safety measures taken by the investigator to protect the study subjects against any immediate hazard, and of any serious breaches of this protocol or of ICH GCP that the investigator becomes aware of.

13. DEFINITION OF END OF TRIAL

This section does not apply to this study.

090177e180ac49ac\1.0\Approved\07-May-2009 14:41

090177e180ac49ac\1.0\Approved\07-May-2009 14:41

14. SPONSOR DISCONTINUATION CRITERIA

Premature termination of this study may occur because of a regulatory authority decision, change in opinion of the IRB/IEC, drug safety problems, or at the discretion of Pfizer. In addition, Pfizer retains the right to discontinue development of PNU-100480 at any time.

If a study is prematurely terminated or discontinued, Pfizer will promptly notify the investigator. After notification, the investigator must contact all participating subjects and the hospital pharmacy (if applicable) within 14 days. As directed by Pfizer, all study materials must be collected and all CRFs completed to the greatest extent possible.

15. PUBLICATION OF STUDY RESULTS

Publication of study results is discussed in the Clinical Study Agreement.

15.1. Communication of Results by Pfizer:

Pfizer fulfils its commitment to publicly disclose the results of studies through posting the results of this study on ClinicalStudyResults.org. Pfizer posts the results of studies that fall into either of the following categories:

- Studies that Pfizer registered on www.clinicaltrials.gov, (ClinicalTrials.gov) regardless of the reason for registration; OR
- All other studies for which the results have scientific or medical importance as determined by Pfizer.

For studies involving a Pfizer product, the timing of the posting depends on whether the Pfizer product is approved for marketing in any country at the time the study is completed:

- For studies involving products already approved in any country and for studies that do not involve a Pfizer product, Pfizer posts results within one year after study completion, defined as Last Subject, Last Visit (LSLV).
- For studies involving products that are not yet approved in any country, Pfizer posts the results of already-completed studies within one year after the first regulatory approval of the product.
- For studies involving products whose drug development is discontinued before approval, Pfizer posts the results within one year after such discontinuation.

Pfizer's posting on ClinicalStudyResults.org includes the following elements:

- Protocol title, study phase, and indication.
- A link to approved product labeling, if applicable.

- The synopsis of study results.
- Citations of known study publications.
- Legal disclaimer.

The study results synopsis posted on ClinicalStudyResults.org (called the PhRMA website synopsis) uses the format established by the ICH-E3 Clinical Study Report (CSR) Synopsis. If posting of study results to ClinicalStudyResults.org jeopardizes a planned publication of the study results, a Pending Full Publication notice is substituted for the synopsis until the study results publication has issued or two years have elapsed, whichever occurs first.

Pfizer posts citations only for publications that are accessible in recognized (searchable) publication databases. Single-centre results publications for a multi-centre study are generally not posted because they may not accurately reflect the results of the study.

15.2. Publications by Investigators

Pfizer has no objection to publication by Investigator of any information collected or generated by Investigator, whether or not the results are favorable to the Investigational Drug. However, to ensure against inadvertent disclosure of Confidential Information or unprotected Inventions, Investigator will provide Pfizer an opportunity to review any proposed publication or other type of disclosure before it is submitted or otherwise disclosed.

Investigator will provide manuscripts, abstracts, or the full text of any other intended disclosure (poster presentation, invited speaker or guest lecturer presentation, etc.) to Pfizer at least 30 days before they are submitted for publication or otherwise disclosed. If any patent action is required to protect intellectual property rights, Investigator agrees to delay the disclosure for a period not to exceed an additional 60 days.

Investigator will, on request, remove any previously undisclosed Confidential Information (other than the Study results themselves) before disclosure.

If the Study is part of a multi-centre study, Investigator agrees that the first publication is to be a joint publication covering all centers. However, if a joint manuscript has not been submitted for publication within 12 months of completion or termination of the Study at all participating sites, Investigator is free to publish separately, subject to the other requirements of this Section.

For all publications relating to the Study, Institution will comply with recognized ethical standards concerning publications and authorship, including Section II - "Ethical Considerations in the Conduct and Reporting of Research" of the Uniform Requirements for Manuscripts Submitted to Biomedical Journals, http://www.icmje.org/index.html#authorship, established by the International Committee of Medical Journal Editors.

090177e180ac49ac\1.0\Approved\07-May-2009 14:41

Publication of study results is also provided for in the Clinical Study Agreement between Pfizer and the institution. In this section entitled Publications by Investigators, the defined terms shall have the meanings given to them in the Clinical Study Agreement.

090177e180ac49ac\1.0\Approved\07-May-2009 14:41

16. REFERENCES

1. World Health Organization. Global tuberculosis control - surveillance, planning, financing: WHO report 2008. WHO report 2008; Geneva; World Health Organization: WHO/HTM/TB/2008.393; 304 pages.

2. World Health Organization. Anti-tuberculosis drug resistance in the world: Report No. 4. WHO report 2008; Geneva; World Health Organization: WHO/HTM/TB/2008.394; 142 pages.

3. Alcala L, Ruiz Serrano MJ, Perez Fernandez Turegano C, et al. In vitro activities of linezolid against clinical isolates of Mycobacterium tuberculosis that are susceptible or resistant to first-line antituberculous drugs. Antimicrob Agents Chemother 2003; 47 (1) :416-7.

4. Cynamon MH, Klemens SP, Sharpe CA, et al. Activities of several novel oxazolidinones against Mycobacterium tuberculosis in a murine model. Antimicrob Agents Chemother 1999; 43 (5) :1189-91.

5. Dietze R, Hadad DJ, McGee B, et al. Early and extended early bactericidal activity of linezolid in pulmonary tuberculosis. Am J Respir Crit Care Med 2008; 178 (11) :1180-5.

6. Condos R, Hadgiangelis N, Leibert E, et al. Case series report of a linezolid-containing regimen for extensively drug-resistant tuberculosis. Chest 2008; 134 (1) :187-92.

7. Fortun J, Martin-Davila P, Navas E, et al. Linezolid for the treatment of multidrug-resistant tuberculosis. J Antimicrob Chemother 2005; 56 (1) :180-5.

8. Park IN, Hong SB, Oh YM, et al. Efficacy and tolerability of daily-half dose linezolid in patients with intractable multidrug-resistant tuberculosis. J Antimicrob Chemother 2006; 58 (3) :701-4.

9. Yew WW, Chau CH, Wen KH. Linezolid in the treatment of difficult multidrug-resistant tuberculosis. Int J Tuberc Lung Dis 2008; 12 (3) :345-6.

10. Nam HS, Koh WJ, Kwon OJ, et al. Daily half-dose linezolid for the treatment of intractable multidrug-resistant tuberculosis. Int J Antimicrob Agents 2009; 33 (1) :92-3.

11. Barbachyn MR, Hutchinson DK, Brickner SJ, et al. Identification of a novel oxazolidinone (U-100480) with potent antimycobacterial activity. J Med Chem 1996; 39 (3) :680-5.

12. Williams KN, Stover CK, Zhu T, et al. Promising anti-tuberculosis activity of the oxazolidinone PNU-100480 relative to linezolid in the murine model. Antimicrob Agents Chemother 2008 :27 pages.

090177e180ac49ac\1.0\Approved\07-May-2009 14:41

13. Wallis RS, Vinhas SA, Johnson JL, et al. Whole blood bactericidal activity during treatment of pulmonary tuberculosis. J Infect Dis 2003; 187 (2) :270-8.

14. Wallis bRS, Palaci M, Vinhas S, et al. A whole blood bactericidal assay for tuberculosis. J Infect Dis 2001; 183 (8) :1300-3.

15. Janulionis E, Sofer C, Song HY, et al. Lack of activity of orally administered clofazimine against intracellular Mycobacterium tuberculosis in whole-blood culture. Antimicrob Agents Chemother 2004; 48 (8) :3133-5.

16. Cheon SH, Kampmann B, Hise AG, et al. Bactericidal activity in whole blood as a potential surrogate marker of immunity after vaccination against tuberculosis. Clin Diagn Lab Immunol 2002; 9 (4) :901-7.

17. Janulionis E, Sofer C, Schwander SK, et al. Survival and replication of clinical Mycobacterium tuberculosis isolates in the context of human innate immunity. Infect Immun 2005; 73 (5) :2595-601.

18. Wallis RS, Song HY, Whalen C, et al. TB chemotherapy: antagonism between immunity and sterilization. Am J Respir Crit Care Med 2004; 169 (6) :771-2.

19. Judd FK, Mijch AM, Cockram A, et al. Isoniazid and antidepressants: Is there cause for concern? Int Clin Psychopharmacol 1994; 9 (2) :123-5.

20. Ogawa Y, Matsumoto K, Kamata E, et al. Effect of feed restriction on the peripheral blood and bone marrow cell counts of Wistar rats. Exp Anim 1985; 34 (4) :407-16.

21. Levin S, Semler D, Ruben Z. Effects of two weeks of feed restriction on some common toxicologic parameters in Sprague-Dawley rats. Toxicol Pathol 1993; 21 (1) :1-14.

22. Gerson SL, Kaplan SL, Bruss JB, et al. Hematologic effects of linezolid: summary of clinical experience. Antimicrob Agents Chemother 2002; 46 (8) :2723-6.

23. Rehm S, White TE, Zahalka EA, et al. Effects of food restriction on testis and accessory sex glands in maturing rats. Toxicol Pathol 2008; 36 (5) :687-94.

24. Loeschke K, Kautz U, Lohrs U. Effects of antibiotics on caecal electrolyte transport and morphology in rats. Klin Wochenschr 1980; 58 :383-85..

090177e180ac49ac\1.0\Approved\07-May-2009 14:41

Appendix 1. Criteria for Safety Values of Potential Clinical Concern

**Hematology**	
Hemoglobin	<0.8 x baseline
Hematocrit	<0.8 x baseline
Leukocytes	<2.5 or >17.5 x $10^3/mm^3$
Platelets	<75 or >700 x $10^3/mm^3$
**Chemistry**	
Total bilirubin	>1.5 times the upper limit of the reference range
AST	>3 times upper limit of the reference range
ALT	>3 times upper limit of the reference range
GGT	>3 times upper limit of the reference range
Alk Phosphatase	>3 times upper limit of the reference range
Creatinine	>1.3 times upper limit of the reference range
BUN/Urea	>1.3 times upper limit of the reference range
Glucose, fasting	<0.6 or >1.5 times the limits of the reference range
Uric acid	>1.2 times upper limit of the reference range
Sodium	<0.95 or >1.05 times the limits of the reference range
Potassium	<0.9 or >1.1 times the limits of the reference range
Calcium	<0.9 or >1.1 times the limits of the reference range
Albumin	<0.8 or >1.2 times the limits of the reference range
Total protein	<0.8 or >1.2 times the limits of the reference range
Creatine Kinase	>2.0 times upper limit of the reference range
**Urinalysis**	
Urine WBC	≥6/HPF
Urine RBC	≥6/HPF
**Vital Signs**	
Pulse Rate	Supine/Sitting: <40 or >120 bpm Erect: <40 or >140 bpm
Blood Pressure	Systolic ≥30 mm Hg change from baseline in same posture
	Systolic <90 mm Hg
	Diastolic ≥20 mm Hg change from baseline in same posture
	Diastolic <50 mm Hg
**Electrocardiogram**	
PR interval	≥300 msec; ≥25% increase when baseline >200 msec;
	Increase ≥50% when baseline ≤200 msec
QRS interval	≥200 msec; ≥25% increase when baseline >100 msec
	≥50% increase when baseline ≤100 msec
QT/QTc interval	≥500 msec

090177e180ac49ac\1.0\Approved\07-May-2009 14:41

Appendix 2. ECG Values of Potential Clinical Concern

**Rhythms of Potential Clinical Concern**
-Asymptomatic Marked Sinus Bradycardia (rate <35 bpm)
-Asymptomatic Supraventricular Couplets, Atrial Bigeminy lasting >30 seconds
-Asymptomatic Ventricular Couplets, Ventricular Bigeminy lasting >30 seconds
-Asymptomatic Type I Second Degree (Wenckebach) AV Block of >30 seconds duration
-Asymptomatic Frequent Premature Ventricular Complexes (PVCs) (≥200/24 hours)
-Asymptomatic Frequent Premature Atrial Complexes (PACs) (≥240/24 hours)
**Adverse Experiences**
-Symptomatic Marked Sinus Bradycardia (rate <40 bpm)
-Asymptomatic Sinus Pause >3 seconds without an escape beat
-Asymptomatic Atrial Flutter or Fibrillation, subcategorized by ventricular response rate: controlled = rate <120 bpm; rapid = rate >120 bpm
-Asymptomatic Supraventricular Tachycardia ≥3 beats (rate >120 bpm)
-Asymptomatic Nonsustained Ventricular Rhythms ≥3 beats, but duration of <30 seconds, including idioventricular rhythm (rate <40 bpm), accelerated idioventricular rhythm (40<x<100) and monomorphic/polymorphic ventricular tachycardia >100 bpm (such as Torsade des pointes)
-Asymptomatic Type II Second Degree (Mobitz) AV Block
-Asymptomatic Complete (third degree) Heart Block
**Serious Adverse Experiences**
-Sustained Ventricular Arrhythmias (>30 seconds duration)
-Ventricular Fibrillation
-At the discretion of the investigator, any arrhythmia classified as an adverse experience

090177e180ac49ac\1.0\Approved\07-May-2009 14:41

Appendix 3. Clinical Protocol Amendment #1

Current Amendment:

Amendment No.	Date	Country (ies)	Site(s)
1	06 May 2009	All	All

Previous Amendments: Not applicable

SUMMARY

090177e180ac49ac\1.0\Approved\07-May-2009 14:41

Reason(s) for Amendment

Depending on the emerging PK data of the parent compound and its metabolites, another PK sample time point at 72 hour will be added in the study. Also, as per the request of FDA, two more PK parameters (CL/F and Vz/F) will be calculated for the parent compound.

The protocol section(s) that have been amended and the details of the changes are summarized in the following sections.

Protocol Section(s) Amended

The protocol sections that were amended are detailed below. The format is as follows:

- The "change from" section represents the current text in the protocol. Bolded text is used to indicate the addition of information to the current text, and strike-out of text (eg, ~~text~~) is used to show the deletion of information from the current text.
- The "change to" section represents the revised text, with the revisions shown in the "change from" section in normal text.

090177e180ac49ac\1.0\Approved\07-May-2009 14:41

090177e180ac49ac\1.0\Approved\07-May-2009 14:41

Schedule of Activities

Change From

**Protocol Activity**	Screen	Day 0	Day 1									Day 2		Day 3		**Day 4[j]**	Follow Up (7-14 days postdose)
**Hours Post Dose**			0 (Predose)	1	2		3	4	6	8	12	16	24	36	48	**72**	
Informed Consent	X																
Admission to Clinical Research Unit		X															
Medical History[a]	X	X															
Physical Examination[b]	X	X													X[c, i]	**X[c]**	X
Height and Weight	X																
Single Supine BP/PR	X		X	X	X		**X**	X		X	X		X	X	X	**X**	X
Hematology	X	X											X		X[i]	**X**	X
Chemistry	X	X											X		X[i]	**X**	X
Urinalysis	X	X											X		X[i]	**X**	X
Urine Cotinine[d]	X	X															
Urine Drug Screen	X	X															
Serum FSH[e]	X																
Triplicate 12-Lead ECG			X	X	X		X	X		X	X		X	X	X[i]	**X**	
Single 12-Lead ECG	X																X
Telemetry[f]			X	X	X		X	X		X							
Study Treatment Administration			X														
PK Blood Sampling			X	X	X		X	X		X	X	X	X	X	X	**X[k]**	
Adverse Event Monitoring			X	X	X			X		X	X	X	X	X	X	**X**	X
Review Prior/Concomitant Medication[g]	X	X															X
Whole Blood Bactericidal Activity Sampling[h]			X		X		X		X		X		X				
Discharge from CRU															X[i]	**X**	

c. Limited physical exam at Period 3 only **for Cohort 1 and Period 4 only for Cohort 2**.

[i] **When not collecting a 72 hour pharmacokinetic analysis sample**

[j.] **If subject is not discharged on Day 3.**

090177e180ac49ac\1.0\Approved\07-May-2009 14:41

[k] **PK sample to be collected for Cohort 1, Period 2 and 3, Cohort 2, Period 1, 2, 3, 4**

Change To

Protocol Activity	Screen	Day 0	Day 1									Day 2		Day 3		Day 4[j]	Follow Up (7-14 days postdose)
**Hours Post Dose**			0 (Predose)	1	2		3	4	6	8	12	16	24	36	48	72	
Informed Consent	X																
Admission to Clinical Research Unit		X															
Medical History[a]	X	X															
Physical Examination[b]	X	X													X[c, i]	X[c]	X
Height and Weight	X																
Single Supine BP/PR	X		X	X	X		X	X		X	X		X	X	X	X	X
Hematology	X	X											X		X[i]	X	X
Chemistry	X	X											X		X[i]	X	X
Urinalysis	X	X											X		X[i]	X	X
Urine Cotinine[d]	X	X															
Urine Drug Screen	X	X															
Serum FSH[e]	X																
Triplicate 12-Lead ECG			X	X	X		X	X		X	X		X	X	X[i]	X	
Single 12-Lead ECG	X																X
Telemetry[f]			X	X	X		X	X		X							
Study Treatment Administration			X														
PK Blood Sampling			X	X	X		X	X		X	X	X	X	X	X	X[k]	
Adverse Event Monitoring			X	X	X			X		X	X	X	X	X	X	X	X
Review Prior/Concomitant Medication[g]	X	X															X
Whole Blood Bactericidal Activity Sampling[h]			X		X		X		X		X		X				
Discharge from CRU															X[i]	X	

c. Limited physical exam at Period 3 only for Cohort 1 and Period 4 only for Cohort 2.

[i] When not collecting a 72 hour pharmacokinetic analysis sample.

j. If subject is not discharged on Day 3.

k PK sample to be collected for Cohort 1, Period 2 and 3, Cohort 2, Period 1, 2, 3, 4

090177e180ac49ac\1.0\Approved\07-May-2009 14:41

Section 2.2; Endpoints

Change From

2nd bullet

- Plasma concentrations of PNU-100480 and its metabolites will be used to determine PK parameters (AUC_{inf}, AUC_{last}, C_{max}, Tmax **and** terminal half-life) as data permit. **Apparent oral clearance (CL/F) and apparent volume of distribution (Vz/F) will also be determined for PNU-100480.**

Change To

- Plasma concentrations of PNU-100480 and its metabolites will be used to determine PK parameters (AUC_{inf}, AUC_{last}, C_{max}, Tmax and terminal half-life) as data permit. Apparent oral clearance (CL/F) and apparent volume of distribution (Vz/F) will also be determined for PNU-100480.

Section 3.1; Study Overview

Change From

Last paragraph

Excluding the screening period for up to 28 days, the study duration for each subject will be up to approximately ~~43~~ **35 day for Cohort 1 and up to approximately 50 days for Cohort 2** ~~of which 15 days will be confined to the CRU (5 days per treatment period)~~.

Change To

Excluding the screening period for up to 28 days, the study duration for each subject will be up to approximately 35 day for Cohort 1 and up to approximately 50 days for Cohort 2.

Section 6.2.1; Day 0

Change From

1st paragraph

Subjects will be admitted to the Clinical Research Unit ~~at least 12 hours~~ **the day** prior to Day 1 dosing and will be required to stay in the CRU for 3 **to 4** days. The following activities will be completed

Change To

Subjects will be admitted to the Clinical Research Unit the day prior to Day 1 dosing and will be required to stay in the CRU for 3 to 4 days. The following activities will be completed

090177e180ac49ac\1.0\Approved\07-May-2009 14:41

Section 6.2.2; Day 1

Change From

7th 8th and 11th bullets

- Collect whole blood for bactericidal activity (Cohort 1, Period 3 only and Cohort 2, Periods 1**, 2** and ~~2~~**3** only, see Section 7.4.1).
- Cohort **2**~~1~~, Period 4 only- subjects should consume high calorie, high fat meal prior to dosing (see Section 4.4.1).
- Assess single supine blood pressure and pulse rate at approximately 1, 2, 3, 4, 8, and 12 hours after dosing.

Change To

- Collect whole blood for bactericidal activity (Cohort 1, Period 3 only and Cohort 2, Periods 1, 2 and 3 only, see Section 7.4.1).
- Cohort 2, Period 4 only- subjects should consume high calorie, high fat meal prior to dosing (see Section 4.4.1).
- Assess single supine blood pressure and pulse rate at approximately 1, 2, 3, 4, 8, and 12 hours after dosing.

Section 6.2.4; Day 3

Change From

1st, 2nd, 4th and 7th bullets

- Limited physical exam (Period 3 only **for Cohort 1 and Period 4 only for Cohort 2 when not collecting a 72 hour pharmacokinetic analysis sample**).
- Obtain triplicate 12-lead ECG measurements approximately 48 hours after dosing on Day 1 **(when not collecting a 72 hour pharmacokinetic analysis sample)**.
- Collect blood and urine samples for safety laboratory tests approximately 48 hours after dosing on Day 1 **(when not collecting a 72 hour pharmacokinetic analysis sample).**
- Discharge from the Clinical Research Unit **(when not collecting a 72 hour pharmacokinetic analysis sample)**.

090177e180ac49ac\1.0\Approved\07-May-2009 14:41

Change To

- Limited physical exam (Period 3 only for Cohort 1 and Period 4 only for Cohort 2 when not collecting a 72 hour pharmacokinetic analysis sample).
- Obtain triplicate 12-lead ECG measurements approximately 48 hours after dosing on Day 1 (when not collecting a 72 hour pharmacokinetic analysis sample).
- Collect blood and urine samples for safety laboratory tests approximately 48 hours after dosing on Day 1 (when not collecting a 72 hour pharmacokinetic analysis sample).
- Discharge from the Clinical Research Unit (when not collecting a 72 hour pharmacokinetic analysis sample).

Section 6.2.5; Day 4

Change From

Section heading and bullets added between last bullet and paragraph of Section 6.2.4

Change To

The following activities will be completed:

- Limited physical exam (Period 3 only for Cohort 1 and Period 4 only for Cohort 2
- Obtain triplicate 12-lead ECG measurements approximately 72 hours after dosing on Day 1.
- Assess single supine blood pressure and pulse rate approximately 72 hours after dosing on Day 1.
- Collect blood and urine samples for safety laboratory tests approximately 72 hours after dosing on Day 1.
- Collect blood samples for pharmacokinetic analysis at 72 hours after dosing on Day 1 (Cohort 1, Period 2 and 3, Cohort 2, Period 1, 2, 3, 4).
- Assess symptoms by spontaneous reporting of adverse events and by asking the subjects to respond to a non-leading question such as “How do you feel?” at 72 hours after dosing.
- Discharge from the Clinical Research Unit.

090177e180ac49ac\1.0\Approved\07-May-2009 14:41

Section 7.1; Blood Volume

Change From

Table 8, Blood Volume

Sample Type	Sample Volume (mL)	Number of Sampling Times			Total Volume (mL)
		Screening	Study Period	Follow-Up	
Safety Labs	7	1	9 (1); 12 (2)	1	77 (1); 98 (2)
PK	~~5~~**4**		3~~3~~**5** (1); 44**8** (2)		1~~6~~**40**~~5~~ (1); ~~220~~**192** (2)
Bactericidal Activity*	3		6(1); 18(2)		18(1); 54(2)
TOTAL**					~~260~~ **235** (1); 3**44**~~72~~ (2)

Change To

Blood Volume

**Sample Type**	**Sample Volume (mL)**	**Number of Sampling Times**			**Total Volume (mL)**
		Screening	Study Period	Follow-Up	
Safety Labs	7	1	9 (1); 12 (2)	1	77 (1); 98 (2)
PK	4		35 (1); 48 (2)		140 (1); 192 (2)
Bactericidal Activity*	3		6(1); 18(2)		18(1); 54(2)
TOTAL**					235 (1); 344 (2)

Section 7.3.1; Plasma for Analysis of PNU-100480 and Metabolites

Change From

1st paragraph

During all study periods, blood samples (**4**~~5~~ mL) to provide a minimum of 2 mL plasma for pharmacokinetic analysis will be collected into appropriately labeled tubes containing K2 EDTA at times specified in the Schedule of Activities.

Change To

During all study periods, blood samples (4 mL) to provide a minimum of 2 mL plasma for pharmacokinetic analysis will be collected into appropriately labeled tubes containing K2 EDTA at times specified in the Schedule of Activities.

090177e180ac49ac\1.0\Approved\07-May-2009 14:41

Section 9.3.2; Derivation of Pharmacokinetic Parameters

Change From

1st paragraph

The pharmacokinetic parameters for PNU-100480, PNU-101603 and PNU-101244 will be determined for each dose of PNU-100480 using non-compartmental methods, as data permit: maximum observed plasma concentration (C_{max}), the time to C_{max} (Tmax), the area under the concentration-time curve from time 0 to the time of the last quantifiable concentration (AUC_{last}), the area under the concentration-time curve estimated from time 0 to 24 hours (AUC_{24}) and infinity (AUC_{inf}), and the terminal phase half-life (t1/2)**, Also, the apparent oral clearance (CL/F) and the apparent volume of distribution (Vz/F) will be calculated for PNU-100480**. PK parameters will be derived from the concentration-time profiles as follows:

Table 10, Pharmacokinetic Parameters

Parameter	Definition	Method of Determination
AUC_{last}	Area under the plasma concentration-time profile from time zero to the time of the last quantifiable concentration (C_{last})	Linear/Log trapezoidal method
AUC_{inf}	Area under the plasma concentration-time profile from time zero extrapolated to infinite time	AUC_{last} + (Clast*/kel), where C_{last}* is the predicted plasma concentration at the last quantifiable time point estimated from the log-linear regression analysis.
AUC_{24}	Area under the plasma concentration-time profile from time zero extrapolated to 24 hours	Linear/Log trapezoidal method
C_{max}	Maximum plasma concentration	Observed directly from data
T_{max}	Time for C_{max}	Observed directly from data as time of first occurrence
t1/2	Terminal elimination half-life	Loge(2)/kel, where kel is the terminal phase rate constant calculated by a linear regression of the log-linear concentration-time curve. Only those data points judged to describe the terminal log-linear decline will be used in the regression.
**CL/F**	**Apparent clearance**	**Dose/ AUC_{inf}**
**Vz/F**	**Apparent volume of distribution**	**Dose/ (AUC_{inf} . kel)**

090177e180ac49ac\1.0\Approved\07-May-2009 14:41

Change To

1st paragraph

The pharmacokinetic parameters for PNU-100480, PNU-101603 and PNU-101244 will be determined for each dose of PNU-100480 using non-compartmental methods, as data permit: maximum observed plasma concentration (C_{max}), the time to C_{max} (Tmax), the area under the concentration-time curve from time 0 to the time of the last quantifiable concentration (AUC_{last}), the area under the concentration-time curve estimated from time 0 to 24 hours (AUC_{24}) and infinity (AUC_{inf}) and the terminal phase half-life (t1/2). Also, the apparent oral clearance (CL/F) and the apparent volume of distribution (Vz/F) will be calculated for PNU-100480. PK parameters will be derived from the concentration-time profiles as follows:

Table 10. Pharmacokinetic Parameters

Parameter	Definition	Method of Determination
AUC_{last}	Area under the plasma concentration-time profile from time zero to the time of the last quantifiable concentration (C_{last})	Linear/Log trapezoidal method
AUC_{inf}	Area under the plasma concentration-time profile from time zero extrapolated to infinite time	AUC_{last} + (Clast*/kel), where C_{last}* is the predicted plasma concentration at the last quantifiable time point estimated from the log-linear regression analysis.
AUC_{24}	Area under the plasma concentration-time profile from time zero extrapolated to 24 hours	Linear/Log trapezoidal method
C_{max}	Maximum plasma concentration	Observed directly from data
T_{max}	Time for C_{max}	Observed directly from data as time of first occurrence
t1/2	Terminal elimination half-life	Loge(2)/kel, where kel is the terminal phase rate constant calculated by a linear regression of the log-linear concentration-time curve. Only those data points judged to describe the terminal log-linear decline will be used in the regression.
CL/F	Apparent clearance	Dose/ AUC_{inf}
Vz/F	Apparent volume of distribution	Dose/ (AUC_{inf}. kel)

090177e180ac49ac\1.0\Approved\07-May-2009 14:41

Section 9.3.3; Statistical Methods

Change From

2nd paragraph, 2nd sentence added

The plasma PK parameters AUC_{inf}, AUC_{24}, C_{max}, AUC_{last}, Tmax and $t_{1/2}$ for PNU-100480 and its metabolite will be summarized descriptively by analyte, cohort, fasted/fed condition, and dose. **Also, CL/F and Vz/F for PNU-100480 will be summarized descriptively by dose and fasted/fed condition. . .**

Change To

The plasma PK parameters AUC_{inf}, AUC_{24}, C_{max}, AUC_{last}, Tmax and $t_{1/2}$ for PNU-100480 and its metabolite will be summarized descriptively by analyte, cohort, fasted/fed condition, and dose. Also, CL/F and Vz/F for PNU-100480 will be summarized descriptively by dose and fasted/fed condition. . .

090177e180ac49ac\1.0\Approved\07-May-2009 14:41
